# Polymer-Based Carriers in Dental Local Healing—Review and Future Challenges

**DOI:** 10.3390/ma14143948

**Published:** 2021-07-14

**Authors:** Dorota Kida, Aneta Zakrzewska, Jacek Zborowski, Małgorzata Szulc, Bożena Karolewicz

**Affiliations:** 1 Department of Drug Form Technology, Wroclaw Medical University, Borowska 211 A, 50-556 Wroclaw, Poland; bozena.karolewicz@umed.wroc.pl; 2 Department of Periodontology, Wroclaw Medical University, Krakowska 26, 50-425 Wroclaw, Poland; aneta.zakrzewska@umed.wroc.pl (A.Z.); jacek.zborowski@umed.wroc.pl (J.Z.); malgorzata.szulc@umed.wroc.pl (M.S.)

**Keywords:** natural and synthetic polymers, multifunctional polymer, polymer drug carrier, polymer material, clinical application

## Abstract

Polymers in drug formulation technology and the engineering of biomaterials for the treatment of oral diseases constitute a group of excipients that often possess additional properties in addition to their primary function, i.e., biological activity, sensitivity to stimuli, mucoadhesive properties, improved penetration of the active pharmaceutical ingredient (API) across biological barriers, and effects on wound healing or gingival and bone tissue regeneration. Through the use of multifunctional polymers, it has become possible to design carriers and materials tailored to the specific conditions and site of application, to deliver the active substance directly to the affected tissue, including intra-periodontal pocket delivery, and to release the active substance in a timed manner, allowing for the improvement of the form of application and further development of therapeutic strategies. The scope of this review is polymeric drug carriers and materials developed from selected multifunctional groups of natural, semi-synthetic, and synthetic polymers for topical therapeutic applications. Moreover, the characteristics of the topical application and the needs for the properties of carriers for topical administration of an active substance in the treatment of oral diseases are presented to more understand the difficulties associated with the design of optimal active substance carriers and materials for the treatment of lesions located in the oral cavity.

## 1. Introduction

An important element determining the progress in the design of new drug carriers is the development of excipients, which, due to their properties depending on therapeutic needs, can enhance the physicochemical properties of the drug formulation, bioavailability, as well as allow the construction of drug formulations with modified or localized release of substances [1]. Excipients are defined as inactive ingredients that are combined with a pharmaceutically active substance to develop a finished drug product for a specific use. Although these substances are on the FDA’s list of inactive ingredients, they generally have well-defined functions in a drug product, i.e., the role of binding, thickening, filler, coating, solubilizing, hydrophilizing, viscosity-inducing, gelling agent, etc. [2]. Modern advances in the technology of drug formulations used for oral application are largely based on the use of macromolecular compounds, which, in addition to the mentioned basic functions of excipients, can perform other additional functions in the drug formulation and exhibit biological activity, e.g., antibacterial, antifungal, immunomodulatory effects, impact on wound, gum and bone healing, or other activities, e.g., sensitivity to stimuli, mucosal properties, etc. [2,3,4]. Polymer excipients for dental applications are a very large and diverse group of excipients, including macromolecular compounds of natural origin, i.e., gelatin, sodium alginate, chitosan, hyaluronic acid, fermentation products, and xanthan gum; semi-synthetic polymers, i.e., cellulose derivatives; and synthetic polymers, i.e., polyethylene glycols, poloxamers, polylactides, polyamides, acrylic acid polymers, etc. [5,6]. This review discusses polymeric drug carriers and drug delivery systems from selected groups of natural and synthetic polymers for the formulation for dental applications. However, taking into account the influence on the therapeutic efficacy of topically administered drug carriers in dentistry, the dynamics prevailing in the oral cavity—including carrier washout by saliva or crevicular fluid in periodontal pockets, the additionally limited time of carrier adhesion to the site of application, and often low penetration of the substance into tissues—a short residence time of the drug formulation at the site of the application is often observed, which reduces the bioavailability of topically administered APIs, failing to reach therapeutic concentrations of the administered drug in the target tissues [7]. New carriers of active substances designed on the basis of polymers guarantee additional therapeutic advantages compared to conventional forms of the drug. These advantages include localization of the action of the substance in the oral cavity to the inflamed area, reduction of systemic side effects of the substance, modification of the duration of action of the drug by obtaining the prolonged or controlled release of the active substance from the carrier, prolonged adhesion of the carrier to the site of application, and additionally the possibility of reducing the frequency of drug dosing, ease of administration, and if necessary, discontinuation of therapy [8,9,10,11].

## 2. Topical Administration of Dental Carriers

The oral cavity, the first segment of the gastrointestinal tract, is the site where many diseases and pathological changes manifest, requiring pharmacological intervention, including topical and systemic therapies [7,12]. However, due to side effects occurring after systemic administration of the drug or low concentration of the active substance reaching the target lesion site, new and improved forms of local administration of active substances with localized effects are still being sought. When designing optimal forms of the drug for topical application, attention should be paid to the specificity and characteristics of the site of administration, i.e., the administration site itself, the size of the administration site, saliva flow, epithelial permeability, etc. [8,13].

### 2.1. Oral Mucosa

The area of the mouth, including the lips, cheeks, dorsal surface and undersurface of the tongue, hard palate, soft palate, alveolar processes of the maxilla and mandible (gums), and the floor of the mouth, is covered by a mucous membrane that is constantly moistened and rinsed with saliva. The total surface area of the mucosa covering the aforementioned areas is about 200 cm^2^ (Table 1), of which 60% is in the mucosa of the cheeks, tongue, and floor of the mouth [14,15]. This membrane consists of stratified squamous epithelium and connective tissue, which are separated by a basal lamina with adherent keratinocytes that multiply rapidly to repair and constantly replenish the epithelium. In some regions of the oral cavity—i.e., attached gingiva, hard palate—the thickness of the epithelium is about 200–250 µm and the cells form layers: basal, spinous, granular, and keratinized. In other areas of the oral cavity, the epithelium does not keratinize, and the cells above the basal layer desquamate. Precisely these areas, i.e., the cheeks or floor of the oral cavity, are the places where, due to the greater permeability of non-keratinized epithelium, the application of drugs is more effective. The thickness of the non-keratinized epithelium is, depending on the location, approximately 100–200 µm in the sublingual region or 500–600 µm for the mucosa covering the cheeks [16]. It is estimated that the permeability of the mucosa is 4 to as much as 4000 times greater than that of the skin [10].

When developing therapeutic preparations for topical use in the oral cavity, it is important to consider other emerging constraints in addition to the site of the application itself, e.g., leaching of the drug carrier by saliva, enzymatic degradation of the active substance, its poor penetration into the tissue, the limited surface area of adhesion of the drug formulation, its unacceptable taste, and possible accidental ingestion of the carrier or specificity of the treated lesion, resulting in lower treatment efficiency. According to the literature, daily saliva production ranges from 0.5 to 1.5 L, and its total flow rate in the absence of stimulation is approximately 0.3–0.4 mL/min. This rate decreases to 0.1 mL/min during sleep and increases to approximately 4.0–5.0 mL/min during eating, chewing, and other stimulating activities [23]. In addition, gingival crevicular fluid, referred to as transudate (or exudate during inflammatory state), is produced in the gingival crevice/periodontal pocket. The rate of secretion and flow of the gingival crevicular fluid depends on the degree of gingival/periodontal inflammation. In healthy subjects, the gingival crevicular fluid flow rate is 3–8 µL/h, in patients with intermediate periodontitis, it is about 20 µL/h, and in a state of advanced periodontitis, it is as high as 137 µL/h. [18].

The penetration profile of the active substance and retention of the used drug carriers after application in the oral cavity should also take into account the mentioned specificity of the treated disease. In Wilson’s lichen and other mucocutaneous diseases, the lesions involve the basal cells of the epithelium and adjacent connective tissue. The immune response occurs primarily in the upper connective tissue. Thus, for a topically applied active ingredient to reach the basal cells, it must pass through the permeability barrier and penetrate deep into the epithelium. In contrast, lesions affecting more superficial epithelial layers, i.e., such as pseudomembranous candidiasis, do not require the drug to cross the permeability barrier [24].

### 2.2. Periodontal Pockets

Other problems in the application of the drug arise in periodontitis, which is a chronic inflammatory process leading to gingivitis and destruction of the alveolar bone and the attachment apparatus of teeth. In the course of periodontitis, so-called periodontal pockets are formed in the oral cavity, which are subgingival spaces filled with dental calculus deposits, bacterial plaque, and granulation tissue as well as lined with non-keratinized inflamed epithelium. Depending on the duration of the disease and the location of the lesions, these pockets vary in size. Their size influences the choice of periodontitis treatment undertaken, which is based mainly on the use of mechanical therapy for pocket cleaning supplemented by the topical application of anti-inflammatory and antibacterial drugs administered directly into the periodontal pockets [25].

### 2.3. Other Needs for Topical Treatment of Oral Diseases

In the surgical treatment of periodontitis, in the presence of bone defects, the patient’s own bone grafts or bone-substitute materials are used for their reconstruction. In order for the implanted materials to be able to integrate into the defect site, they must be isolated from the epithelium that is rapidly growing into the surgical site. Barrier membranes are used to inhibit epithelial tissue from growing into the surgical site; when placed over the implanted material, under the gingival flap, they are semi-permeable and allow the area around the implant to heal. Traditionally used barrier membranes in guided tissue regeneration and/or guided bone regeneration (GTR, GBR) procedures are created from animal-derived collagen and, with advances in technology, also from biodegradable polymers. In addition to their epithelial isolating properties, barrier membranes can also play a therapeutic role if an active substance with a controlled release is incorporated into their structure [26,27].

In addition, some oral procedures or disease symptoms require the administration of local anesthesia to the mucosa or treatment of oral mucositis with lidocaine hydrochloride, benzydamine hydrochloride, opiates, and amylmetacresol/dichlorobenzene alcohol. It should be mentioned that more than 50% of patients treated for head and neck cancer suffer from oral mucositis and damage to the oral epithelium, leading to painful inflammation and ulceration, which is caused by ongoing chemotherapy or radiotherapy [28]. The traditional administration of anesthesia involves an injection, which makes the patient fearful and reluctant to the visit, even though after the injection, treatment pain is completely or largely eliminated [29]. Topical forms of anesthesia administration are convenient to use and reduce anxiety and pain for the patient. However, they are not always adapted to the specific structure of the oral cavity. A spray, cream, or gel with an anesthetic substance does not stay on the mucosa long enough, which reduces the chance of the anesthetic reaching the nerve endings or leads to its duration is not lasting. Therefore, for use in patients with moderate pain or for minimally invasive procedures i.e., scaling, carriers that last longer at the site of administration are being used, allowing the anesthetic effect to be achieved and sustained for the intended time [30].

## 3. Carriers and Biodegradable Polymeric Materials in Dentistry

### 3.1. Natural Polysaccharides

Polysaccharides, which are important natural biological macromolecules, can be defined as polymeric carbohydrate structures composed of repeating monosaccharide units linked by glycosidic bonds [31]. In this paper, drug dosage forms based on chitosan and its derivatives, hyaluronic acid and its derivatives, and pectins and natural gums were characterized for the purposes of application as excipients in the technology of drug carriers for oral application (Table 2).

#### 3.1.1. Chitosan and Its Derivatives

**Figure 1:**
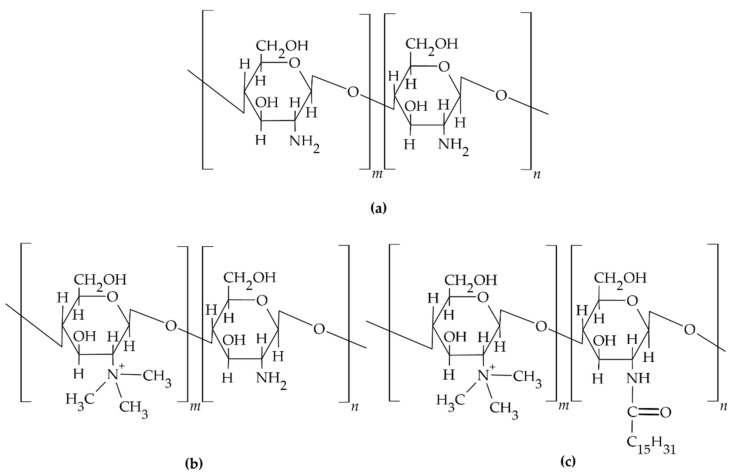
Molecular structure of the chitosan repeated monomer unit (**a**), chitosan derivatives: trimethyl chitosan (**b**), N-trimethyl chitosan-g-palmitic acid (**c**) [52].

Chitosan is a naturally occurring biodegradable, biocompatible polysaccharide in mammalian cells; it is a copolymer of D-glucosamine and N-acetyl glucosamine that is mainly obtained by the deacetylation of chitinous exoskeletons of various crustacean species [50]. The structural formula of the chitosan molecule is shown in Figure 1. In the linear molecule of chitosan, hydrogen bonds are formed between hydroxyl and amino groups, which influences the structure and flexibility of the polymer chain. In addition, the amino groups of the polymer chain are protonated to form salts with inorganic and organic acids, i.e., acetates, citrates, hydrochlorides, and lactate. By appropriately selecting the molecular weight and degree of deacetylation of the polymer, varieties with different physicochemical properties are obtained. These properties determine the choice of polymer for the design of dental carriers obtained with the use of chitosan, e.g., matrices, films, nanoparticles, or semi-solid dosage forms with the desired pharmaceutical properties [51]. There are varieties of the polymer with different molecular weights in the range of 3800–2,000,000 and degrees of acetylation from 40% to 98%.

In research conducted to date, the biological, antibacterial, antifungal, and immunomodulatory activities of chitosan and chitooligosaccharides have been confirmed [50,53]. In addition, the literature indicates that chitosan used in biocompatible dental media accelerates periodontal tissue regeneration, showing both the mentioned antimicrobial [54] and anti-inflammatory effects [55]. This activity is due to the interaction between the positively charged amino group of the glucosamine molecule and the negatively charged components located on the surface of the G-negative cell membrane of microorganisms. The properties of the cell membrane of the bacteria are altered, the transport of nutrients to the inside of the cell is reduced, and its contents leak out. Another possible explanation for the aforementioned polymer activity is that chitosan penetrates the bacterial cell and binds to DNA, which then causes an inhibition of RNA transcription and thus protein synthesis. This process depends on the physicochemical properties of the polymer, its molecular weight/degree of polymerization and degree of deacetylation, as well as on the type of microorganism. The relationship between the number of acetylated groups, the structure of the polymer, and the mechanism of its antimicrobial activity has not been clearly established on the basis of the studies conducted, and it has not been indicated which property of the polymer is crucial for its biological activity [56,57,58]. However, chitosan is known to exhibit potent activity against oral plaque-forming pathogens, in particular: *Porphyromonas gingivalis*, *Prevotella intermedia*, and *Aggregatibacter actinomycetemcomitans* (formerly *Actinobacillus actinomycetemcomitans)* [50].

In the design of chitosan-based drug carriers applied to the oral mucosa, the mucoadhesive properties of the polymer are important, allowing the drug dosage form to retain and prolong its residence time at the site of administration, ensuring a high concentration gradient of the released active pharmaceutical ingredients [12]. Chitosan is a cationic polymer whose mucoadhesive properties result from electrostatic interactions between the positive charge of the polymer’s amino groups and the negatively charged sialic acid residues of the mucus glycoproteins [59,60,61]. The potency of the interaction of chitosan-based engineered carriers with the mucosa depends on the number of available amino groups of the polymer reacting with the negatively charged groups of the epithelium, as well as on the charge density, molar mass, degree of acetylation, and flexibility of the polymer chain and its conformation [9,11,62,63]. With the increase in molar mass of the polymer and the ability to penetrate deeper mucin layers by the polymer chains, the mucoadhesion strength of the polymer also increased [64]. In addition, the work of Roldo et al. [65] found that a medium molecular weight polymer exhibits a higher mucoadhesion capacity than the low and high molecular weight varieties. Chitosan, in addition to its mucoadhesive properties, has the property of loosening the connections between adjacent mucosal epithelial cells and, due to its ability to create desmosomes, improves the transport of active substances across biological membranes [66]. The described property of the polymer was confirmed during in vitro testing using the intestinal epithelium model, where the effect of 0.5–1.5% chitosan solutions on increased permeability of the cell layer of the Caco2 cell line to hydrophilic macromolecular substances, i.e., mannitol and dextran, was demonstrated [67].

In a clinical study, Atai et al. evaluated the efficacy of a new liquid antifungal formulation derived from low molecular weight chitosan with a molecular weight of 5000 Da in the treatment of prosthetic stomatitis caused by *Candida albicans* colonization. A randomized, single-blind clinical trial evaluating the application of a rinse containing a suspension of 100,000 U/mL of nystatin in a 1% polymer solution, pH 5.0, in a group of 40 patients, confirmed a significant reduction in erythematous area, a reduction in burning sensation, and a reduction in the number of blastospores in the fungus-infected area of the palatal mucosa, while also shortening the time taken to achieve clinical improvement. The demonstrated antifungal efficacy of chitosan makes it a promising active ingredient for mouthwashes [68].

In their study, Özdogan et al. obtained 2% gels based on two types of chitosans with atorvastatin: an alkaline variety in 1% lactic acid solution and a water-soluble variety, ultimately for use in the treatment of inflamed periodontal pockets after local application with a syringe. In vitro studies found that the formulations exhibited adhesive properties suitable, according to the researchers, for maintaining the carrier at the site of application and, importantly, for eliminating the phenomenon of rapid washout caused by saliva after application. Furthermore, the release of atorvastatin from the gels was found to be slower compared to the release of atorvastatin from a reference solution prepared with polyethylene glycol 400 (PEG 400). In vitro studies on human gingival fibroblast cells induced by tumor necrosis factor (TNF)-alpha found that the release of pro-inflammatory cytokines decreased after treatment with atorvastatin gels, and the concomitant presence of chitosan in the formulation enhanced the anti-inflammatory effect of the carrier. At the same time, in the evaluation of the anti-inflammatory effect of atorvastatin gels, no clear difference was observed between the use of the two polymer varieties, alkaline chitosan and water-soluble chitosan [33].

Pignatello et al. developed a chitosan glutamate and glycerin-based mucoadhesive hydrogel containing 5% lidocaine hydrochloride for oral mucosal applications. The anesthetic effect of mucoadhesive hydrogels was evaluated in vivo after application to the buccal mucosa and compared with the commercial semi-solid preparation Xylocaina^®^ gel (Astra Zeneca S.p.a., Basiglio, Italy) containing 5% of the substance. Clinical evaluation has confirmed the beneficial potential of the formulation in minimizing pain associated with oral mucosal diseases, indicating that it could be used to alleviate symptoms of aphthosis or other painful oral diseases [36].

Another clinical study evaluated the effect of a topically applied chitosan-based gel with 0.2% chlorhexidine, allantoin, and dexpanthenol (Bexident Post, ISDIN, Spain, Barcelona) on post-extraction wound healing in 50 patients with symmetric extractions. Patients were divided into study and control groups, both consisting of 25 patients. The study group, after application of the chitosan-based formulation with active substances, did not show infectious wound complications in the postoperative period, while faster healing of lesions was observed. This pointed to the antimicrobial, regenerative, and hemostatic effects of the carrier and highlighted the mucoadhesive properties of the formulation resulting from the presence of cationic chitosan [53,69].

A chitosan-based gel formulation was also studied by Lopez et al., where its effectiveness in controlling postoperative inflammation, pain, and healing in patients undergoing molar extraction was compared to a bicarbonate-based oral rinse. Two groups of 24 patients each underwent symmetric removal of retained lower third molars. Postoperatively, a bicarbonate rinse was used 3 times a day (1 tablespoon dissolved in 200 mL of water) in one group and the aforementioned gel was used in the other. In patients undergoing dental procedures, the chitosan-based gel product was found to be more effective than the rinse in controlling inflammation and pain, which was assessed daily on a VAS scale for 7 days after the procedure. As a result, the use of the gel reduced the use of analgesics, promoting better wound scarring [21].

Other studies in periodontitis patients evaluated the effect of chitosan-based gels on periodontal tissue regeneration. Twenty patients underwent oral hygiene instruction and a scaling and root planing (SRP) procedure. After 8 weeks of initial therapy, patients were divided into 4 groups. Each group was treated for bone defect of the alveolar process by surgical flap procedure and removal of granulation tissue. In addition, 1% chitosan gel was applied to the defect in group A, an analogous 1% chitosan gel with demineralized bone matrix (DBM) was applied to the defect in group B, chitosan gel with collagen membrane was applied to the defect in group C, and only a flap procedure was performed in group D, which was also the control group. Clinical and radiological evaluation was performed 90 and 180 days after the procedure. In radiographic evaluation, better regenerative effects were obtained in all groups treated with chitosan gels compared to control group D. However, no significant differences were observed between groups A, B, and C treated with different chitosan formulations [70].

By modifying the electrostatic interactions originating from the amino group in the chitosan molecule, thermosensitive carriers with extended contact time and release of the incorporated drug at the application site are obtained. Pakzad et al. [35] obtained a semi-solid thickening formulation based on chitosan and glycerolphosphate with gelatin, forming a gel in the physiological temperature range of 37 °C, which allowed in vitro prolonged release of metronidazole from the developed hydrogel over 8 days. The change in viscosity of the formulation as a result of the sol–gel transition was explained by the formation of electrostatic interactions between the positively charged amino groups of chitosan and the negatively charged phosphate residues of β-glycerolphosphate and the formation of hydrophobic and hydrophilic bonds between the polymer chains. Thermosensitive carriers with controlled gelation time and prolonged release of the active substance are a promising prospect for the development of drug formulations for low molecular weight substances applied both directly to the periodontal mucosa and areas with limited possibility of carrier administration, i.e., the periodontal pocket [35,43,71].

In another study, Wu et al. assessed a chitosan-β-glycerophosphate (CS/β-GP)-derived sol–gel hydrogel system releasing vascular endothelial growth factor (VEGF) and further investigated its effect on dental pulp stem cells (DPSCs) from the extracted retained molars. DPSCs cultured in CS/β-GP hydrogel maintained adherence and viability, and the results suggest that VEGF-loaded CS/β-GP hydrogel may continuously release vascular endothelial growth factor and contribute to odontogenic differentiation of DPSCs. Additionally, when evaluated, the CS/β-GP hydrogel with VEGF promoted odontogenicity and the differentiation of DPSCs to a greater extent than VEGF applied without the hydrogel carrier. The results suggest that the chitosan-based hydrogel could become a potential carrier for bioactive molecules used in capping and coating therapy of living dental pulp [32].

In a study by Uysal et al., the efficacy of a chitosan toothpaste (AloeDent) in inhibiting enamel demineralization around orthodontic brackets compared to a conventional non-fluoridated toothpaste (Sensodyne Mint) was evaluated in sixteen orthodontic patients. It has been shown that the use of a tooth cleaner containing chitosan can reduce enamel decalcification occurring in patients with inadequate oral hygiene [72]. A similar evaluation was also performed by Schlueter et al. in a study group of 10 patients, in which the use of the F/Sn-containing toothpaste (1400 ppm F^−^, 3500 ppm Sn^2+^) and F/Sn/chitosan toothpaste (1400 ppm F^−^, 3500 ppm Sn^2+^, 0.5% chitosan) was compared to the placebo toothpaste as a negative control and the SnF_2_-containing gel as a positive control (3000 ppm Sn^2+^, 1000 ppm F^−^). The F/Sn/chitosan formulation proved more effective than the F/Sn-containing toothpaste (*p* ≤ 0.001), and its efficacy against erosive and erosive-abrasive tissue loss was also confirmed. This study suggests that using a toothpaste containing F/Sn/chitosan could also provide good protection for patients who frequently consume acidic foods [73].

Based on chitosan, Freitas et al. obtained mucoadhesive films containing the topical anesthetic spilanthol, which is present in an ethanol extract from the *Acmella oleracea* plant also known as the ‘Toothache Plant’. The developed solid carriers showed high in vitro penetration of the active substance through the mucosa of the esophageal epithelium of a pig, and the efficacy of anesthetic action through in vivo studies in a mouse animal model, which is comparable to the reference EMLA^®^ cream containing a eutectic mixture of lidocaine and pilocarpine (Aspen Pharma Trading Limited, Ireland) applied to the skin and mucous membranes. A mucoadhesive film containing 10% extract of the *Acmella oleracea* plant treated with activated charcoal is a potential alternative to the oral topical administration of anesthetics, encouraging further clinical trials. In addition, the ethnopharmacological uses of this plant species, their safety, and their low toxicity indicate that the developed carrier could be an alternative to topical anesthetics currently available in dentistry [20].

In another study, Khajuria et al. found that after the administration of a mucosal film based on chitosan with metformin hydrochloride, there was a reduction in the number of periopathogens, i.e., *P. gingivalis* and *Tannerella forsythia* under in vitro conditions. In application tests of the said bioabsorbable film with metformin on a rat model, regeneration of the alveolar process lost in the course of induced periodontitis was favorably evaluated [37].

Using a gel freezing process with solvents such as acetic acid or ascorbic acid, Qasim et al. prepared chitosan-based hydroxyapatite-containing porous non-resorbable membranes for GTR administration. The acid chosen for chitosan dissolution determined the physicochemical properties of the designed membranes, i.e., the size and structure of the obtained pores, their swelling coefficient, and their degradation rate. It was observed that the use of ascorbic acid to dissolve chitosan resulted in a more uniform pore distribution in the membrane compared to the use of acetic acid. Membrane-derived culture of human osteosarcoma cells (MG63) and human embryonic-derived mesenchymal progenitor cells (hES-MP) showed that the resulting structures supported cell proliferation, thus confirming their potential utility for periodontal tissue regeneration applications [40]. In subsequent studies using the electrospinning technique, the aforementioned author obtained chitosan-based nanofibers with a 5% addition of polyoxyethylene for further use as a surface layer in GTR membranes for administration in periodontal tissue regeneration. Three-dimensional mechano-resistant fibrous membranes with arbitrary or ordered nanofibers orientation were obtained. The results of the study indicated that both orientations can mimic the local structure and function of a specific tissue in the regeneration process of a periodontal defect site. With an ordered orientation of nanofibers in the membrane, it can mimic ligaments. With an arbitrary arrangement of nanofibers in the structure, it can mimic bone tissue, improving the regeneration of osteoblastic cells at the site of a jawbone defect [41].

Chitosan and chitosan derivatives, i.e., trimethyl chitosan and N-trimethyl chitosan-g-palmitic acid (Figure 1b,c), have also been used to design multicomponent microsphere with controlled release of the active pharmaceutical ingredient [50,60]. In the work of Yadav et al., vanillin cross-linked microspheres, <50 µm in diameter, containing ornidazole and doxycycline were obtained from chitosan to treat periodontal pockets. Vanillin, a natural covalent cross-linking agent, was used to create linkages between the hydroxyl groups of the phenolic aldehyde that make up the vanillin structure and the amino groups of the chitosan for a prolonged and controlled release of APIs. From the obtained chitosan microspheres suspended in a thermosensitive carrier based on Carbopol/Pluronic P127/Pluronic P68 polymers, prolonged release of both incorporated substances was confirmed during in vitro tests over the duration of 7 days. A biphasic release profile was observed for both ornidazole and doxycycline. After a rapid release phase lasting about 2 days with part of the drug dose adsorbed on the microsphere surface and dispersed inside the hydrogel, a phase of slow controlled release of API resulting from erosion of the microsphere matrix was observed. Higuchi’s equation described the release process of both studied APIs. The calculated value of diffusion coefficient n > 0.5 confirmed that the mechanism of drug release was different from Fick’s diffusion law. The polymeric matrix diffusion and swelling controlled the release. In a clinical study conducted in a group of 10 patients with moderate to severe periodontitis and at least three pockets ≥ 5 mm deep, the efficacy and therapeutic potential of injectable designed carriers were evaluated. In addition, their effects on plaque index (PI), gingival index (GI), bleeding of probing (BoP), pocket probing depth (PPD), and clinical attachment loss (CAL) at 15, 30, and 60 days after the start of treatment were investigated. The evaluated microspheres in a gel carrier administered inside of the pockets showed a significant reduction in clinical parameters including BoP, GI, and PI when compared to sole scaling and root planing (SRP) treatment at 30 and 60 days after administration [22].

Govender et al. in a precipitation process from a solution containing 3% chitosan, 9% tripolyphosphate, and 10% tetracycline hydrochloride obtained microspheres for which they carried out an evaluation, i.e., morphology, surface pH, hydration dynamics, thermal properties, and analysis of the release kinetics of the carrier and its antimicrobial activity. Interpretation of the release of the substance from chitosan microspheres using kinetic models indicated that the release of tetracycline proceeded according to Fick’s diffusion law, while texture analysis showing the hydration dynamics indicated minimal wetting of the carriers during the 8 h study. It was evaluated that the outer porous structure of the microspheres attracts water and the highly cross-linked inner structure resists water ingress, thus contributing to reduced hydration. The antimicrobial activity of the obtained microspheres was tested using the gradient-diffusion method, which showed that the amount of substances released from the in vitro carriers exceeded the MIC value required to inhibit the growth of the *Staphylococcus aureus* strain isolated from inflamed periodontal pockets. The results regarding the physicochemical characteristics, bioadhesion, and release kinetics of tetracycline from microspheres confirmed their potential to be carriers for the active substance in topical therapy of periodontal diseases. Given the size of the carriers, they can penetrate mucosal areas located below the gingival line, which are in practice inaccessible to other conventional drug dosage forms [42].

Pichayakorn and Boonme received chitosan-based microparticles with incorporated metronidazole (MTZ-MP) during an emulsion cross-linking process. In their study, the authors compared the in vitro release of MTZ from hydrogels and films containing MTZ-MP and free substance (MTZ). Gels into which microparticles with metronidazole were introduced were obtained by cross-linking glutaraldehyde with a 3% chitosan solution and films by evaporation from a 2% cross-linked chitosan solution. It was observed that hydrogels with both MTZ-MP and MTZ provided constant release rates over the tested 6 h period, while films had a rapid release of MTZ in the first 5 min of the test, after which the release curve reached a plateau phase. The migration of the substance during its preparation by solvent evaporation explained the phenomenon of rapid release of MTZ from the films to the surface of the carrier. As a drawback of the developed microparticles, the researchers pointed out the possible toxicity of glutaraldehyde used as a cross-linking agent, which needs additional evaluation in the next planned stage of work [43].

Shuo et al. used polyelectrolyte complexes of chitosan and carboxymethyl–chitosan nanoparticles prepared during ionotropic gelation as carriers for doxycycline. In the in vitro evaluation on cell lines, the authors confirmed the bacteriostatic effect of the nanocarriers against *P. gingivalis* (ATCC 3327). In a subsequent in vitro study using human gingival fibroblast cells (HGFs), obtained during alveolar surgery from inpatients, they demonstrated the effect of the carriers on reducing levels of mRNA, IL-1B, and NLRP3 (NAcht Leucine-rich repeat Protein 3). The favorable results obtained in this study require further clinical evaluation, providing further opportunities for the application of the drug in chitosan-based nanocarriers for the treatment of periodontal disease [74].

**Figure 2:**
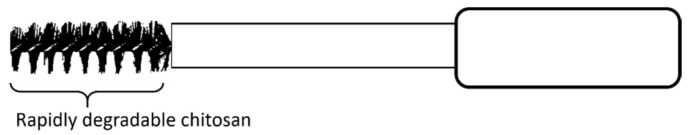
Single-use dental device with a working tip of rapidly degradable chitosan attached to a medical-grade stainless steel stem covered with a white soft polypropylene sleeve. A device used with an oscillating dental tip (average 600–1000 rpm) for cleaning up to four dental implants with peri-implant mucositis in the patient.

The use of chitosan in dentistry also includes the development of implant cleaning brushes. A 6-month split-mouth-design pilot randomized clinical trial involving 11 patients with a total of 24 dental implants who were diagnosed with peri-implant mucositis compared the efficacy of brushes (Figure 2) with a fast-degrading polymer working tip (Labrida BioClean^TM^, LABRIDA AS, Oslo, Norway) and an oscillating dental handpiece (ER10M, TEQ -Y, NSK Inc., Kanuma Tochigi, Japan) to scaling procedures involving titanium curettes (Langer and Langer, Rønvig, Denmark). Implants were randomly assigned to either treatment with a chitosan brush using an oscillating dental handpiece or treatment with titanium curettes. All treated areas showed reduced signs of inflammation 6 months after implementation of baseline treatment and 3 months after the implementation of maintenance treatment. Patients whose implants were treated with a chitosan tip showed a greater improvement in bleeding rates during BoP than patients treated with titanium curettes. Thus, a chitosan-based brush also appears to be an effective tool for cleaning dental implants [75].

#### 3.1.2. Hyaluronic acid (HA) and Hyaluronate

HA is a component of the extracellular matrix found in the skin of connective, epithelial, and nervous tissue. It is also a key building block of the periodontal ligament structure and the connective tissue of the gums. HA is a biocompatible, bioresorbable, anionic glycosaminoglycoside, with each disaccharide moiety composed of d-glucuronic acid and d-N-glucosamine, which are linked by alternating β-1,4 and β-1,3 glycosidic bonds [76]. The length of the sugar chain, the molecular weight, and the concentration of the polysaccharide affect its physical and physiological properties, i.e., its viscosity and viscoelastic properties, which are related to the function of the build tissues. Under physiological conditions, the presence of bacterial hyaluronidase and low molecular weight HA in gingival tissues is an important signal for the development of periodontitis, mobilizing the immune system. In turn, the presence of high molecular weight HA attests to the proper functioning of tissues, thereby limiting inflammation and suppressing the immune response. In addition, HA as one of the most hydrophilic natural molecules regulates the osmotic pressure of cells and, by absorbing significant amounts of water, ensures optimal tissue hydration, thus playing a key role in the correct course of tissue repair and regeneration [4].

**Figure 3:**
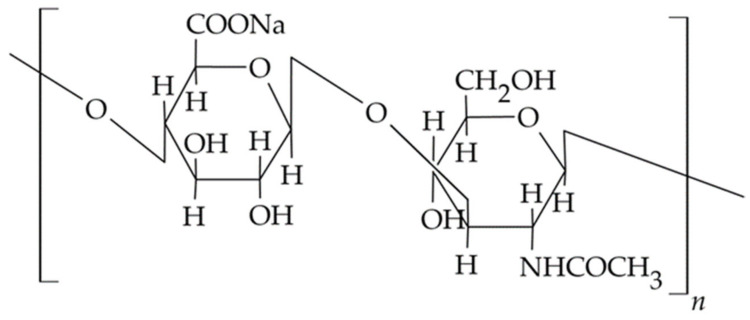
Molecular structure of HA molecule [79].

The HA molecule shown in Figure 3 contains carboxyl functional groups, primary and secondary alcohols, and N-acetyl groups, whose modification by chemical or physical means allows obtaining polymer derivatives with desired physicochemical properties and biological activity. In an aqueous medium, hydrogen bonds between adjacent carboxyl and N-acetyl groups of polymer subunits maintain the rigidity of the polymer network with a double helix structure, which enables the diffusion and retention of significant volumes of water in the formed structure. The study shows that hyaluronate chains occupy a large hydrodynamic volume in aqueous polymer solution already at concentrations in the range of 3–5 mg/mL; thus, they fill the solvent space. Such an arrangement creates a selective barrier for the molecules of the incorporated substance, in which small molecules can diffuse freely, while larger molecules are partially or completely stopped, which affects the diffusion of the drug from polymer-based carriers [77,78].

Topically administered drug dosage forms of HA and hyaluronate are recognized as complementary treatments for periodontitis, improving and accelerating wound healing and tissue regeneration [78,80,81]. HA applied to the wound in the form of a viscous elastic gel reduces the penetration of bacteria and viruses into the tissues while showing bacteriostatic, fungistatic, immunomodulatory, anti-inflammatory properties, as well as accelerating mesenchymal stem cell differentiation and dentin regeneration [82,83]. Topical application of HA in a concentration of 0.2% in the form of liquid, gel, or spray in the treatment of gingival inflammation caused by the accumulation of plaque and periodontitis has a beneficial effect on improving clinical parameters and reducing the values of indicators such as the clinical Periodontal Index (PI), the Papillen–Blutungs Index (PBI), the Sulcus Bleeding Index (SBI), or improvement of variable Gingival Crevicular Fluid (GCF) parameters and reduction of periodontal pocket depth [4,84,85,86,87]. The gel polymer is also used as a filler in the correction of soft tissue defects in the minimally invasive non-surgical method of gingival papilla reconstruction in the interdental spaces. In a comparative study, a group of patients who used HA gel for papilla reconstruction in relation to a control group where only saline solution was used highly rated the aesthetic results achieved even 6 months after application of the formulation [88]. Similarly, favorable results have been obtained after the use of polymer gel in procedures aimed at covering gingival recessions [89]. The results of the study in this case showed a statistically significant improvement in the effect of coverage of the gingival recession using the polymer gel, with the gel layer also accelerating the healing process by mechanically isolating and protecting the treatment site. This property of polymer-based carriers has also been used successfully in the topical treatment of recurrent aphthae [90], Wilson’s lichen [91], or oral candidiasis. Reports in the literature have also confirmed the efficacy of HA-based gels in the treatment of peri-implant inflammation, particularly in the early stages of disease development, suggesting its protective effect in reducing bacterial colonization around the implant [92]. In the study by Hosny et al., a nanoemulsion with miconazole nitrate was introduced into a HA-based gel to obtain a nanoemulgel. During in vitro studies, this formulation was found to improve the solubility of the encapsulated substance further, facilitating the penetration of miconazole into tissues and allowing for a prolonged release of the substance at the application site. The end result is that the nanoemulgel form provides better efficacy in oral candidiasis therapy and reduces the frequency of dosing [93]. In addition, 0.8% HA-based gel with platelet-rich fibrin (L-PRF) has been used in other surgical procedures, including furcation treatment [94], which also improved the clinical results in terms of reduction of inflammation and reduction of bone loss in the area of teeth furcation. This combination of HA and fibrin clot facilitates the infiltration of extracellular matrix cells into the site of inflammation and induces, through fibroblasts, cementoblasts, osteoblasts, and among others, the production of pro-inflammatory cytokines. In the subsequent phase of wound healing, HA promotes cell proliferation and reorganization of granulation tissue. Thanks to hyaluronidase, HA depolymerizes, with the lower molecular weight polymer facilitating angiogenesis and the differentiation of mesenchymal cells into osteoblasts [95].

Fujioka et al. in an in vitro study evaluated an HA-based carrier containing recombinant human bone morphogenetic protein 9 (rhBMP9), which is one of the most osteogenic growth factors of the BMP family, confirming the beneficial properties of the carrier as a potential formulation for said protein [46]. The rhBMP9 factor adsorbed in the spatial structure of cross-linked butanediol diglycidyl ether (BDDE) HA was released slowly and in a controlled manner, prolonging its osteogenic effect concerning pure protein administration. In addition, the polymer alone showed the ability to more than double the induction of osteoblast differentiation by increasing mRNA levels and increasing the expression of osteocalcin (OCN), which is a late marker of osteoblast differentiation, while the combination of HA with rhBMP9 induced up to a fourfold increase in osteoblast differentiation. The above study confirmed the advisability of using cross-linked HA as a carrier for growth factors in tissue engineering and, at the same time, as a formulation ingredient to support osteogenesis [96,97]. In the study by Afat et al. [45], a porous HA-based matrix with incorporated platelet-rich fibrin (Leukocyte-Platelet Rich Fibrin, L-PRF), obtained from centrifuged patient blood, was introduced into the dental alveoli after extraction of third molars. After matrix application, a significant reduction in postoperative complications alongside a shorter wound healing time was observed compared to a control group of patients whose post-extraction dental alveoli were left to heal spontaneously. HA has also been successfully combined with synthetic bone substitute, and the resulting combination has been used in procedures such as maxillary sinus lift [98], socket preservation after tooth extraction, or regeneration of bone defects to increase bone density [99]. HA can also be used as a biocompatible matrix in tissue engineering for stem cells and signaling molecules to reconstruct the temporomandibular joint, salivary glands, dental pulp, and other dental tissues [100,101,102]. Such use of the HA matrix offers favorable prospects in the development of regenerative medicine for the treatment of conditions and injuries in dentistry.

Factors limiting the use of HA as a carrier for active substances include its low mechanical resistance and possible rapid degradation in tissues caused by hyaluronidase. This phenomenon can be reduced by using combinations of HA with other polymers, e.g., sodium alginate, in the formulation or by obtaining spatial biomaterials with limited enzyme exposure [83,103]. In the work conducted by Li et al., hemostatic films, based on the conjugation of obtained HA with pullulan, were prepared to accelerate the healing of superficial traumatic wounds. The obtained carriers were characterized by a spatial structure formed into a network and a larger diameter of pores and water sorption capacity compared to dressings obtained from these polymers without modification. The dressing with higher sorption capacity absorbed more fluid, preventing the accumulation of exudate inside the wound and at the same time reducing the frequency of its replacement. In order to reduce enzymatic degradation, improve mechanical properties, and extend the residence time of HA-based hydrogel carriers in the application site, attempts were made to modify them by amination and oxidation with genipin, cross-linking with polyethylene glycol diepoxide, diamine, or linking with methacrylate derivatives. The obtained hydrogels showed a slower degradation with respect to unmodified HA in the presence of hyaluronidase, but the toxicity of cross-linking agents and biocompatibility of the obtained materials have not yet been evaluated in this study [31]. Park et al. developed HA-adipic acid conjugate-coated PLGA barrier membranes with controlled degradation time for use in periodontics with significant bone defects. The membrane barrier caste was obtained by the chemical modification of HA using adipic acid dihydrazide, leading to an amide bond between the components. The developed polymer film was biocompatible and enzyme-resistant up to 8 weeks after application in an animal model. In an in vivo study, 60% regeneration of the bone defect was achieved within 12 weeks of covering it with the prepared film [44].

#### 3.1.3. Gums and Pectins

**Figure 4:**
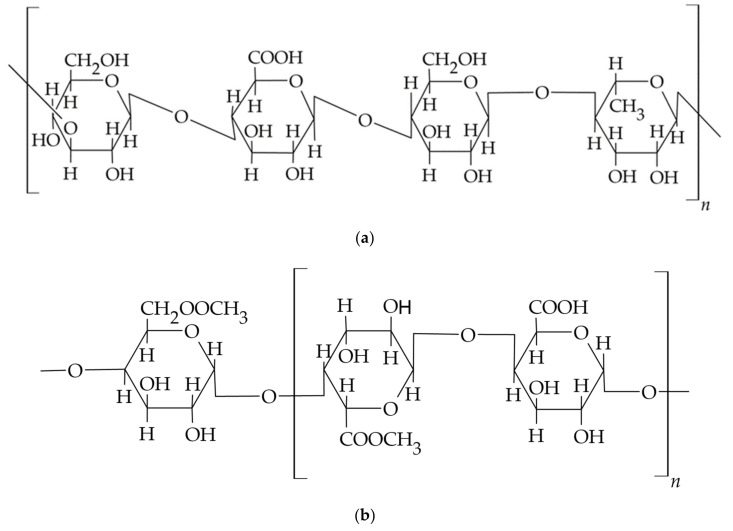
Molecular structure of gellan gum (**a**) and pectin (**b**) [104].

Both gums, i.e., gellan and karaya, and pectins are biodegradable and biocompatible natural polysaccharides used in drug formulation technology as excipients to increase the viscosity of aqueous solutions, produce hydrocolloid dispersions of active ingredients, prepare gels, or serve as thickening agents to develop solid swelling carriers [104]. Gellan gum is an example of an anionic, water-soluble exopolysaccharide derived from carbohydrates fermented by the bacteria *Pseudomonas elodea* and used to develop carriers in dentistry. The polymer molecule shown in Figure 4a has a linear structure consisting of a backbone of a repeating unit of β-d-glucose (d-Glc), l-rhamnose (l-Rha), and d-glucuronic acid (d-GlcA) and two acyl groups, acetate and glycerate, bound to the glucose residue adjacent to the glucuronic acid. Two native forms of gellan gum are available, acetylated and deacetylated, and both forms of the polymer have the ability to form thermosensitive gels with different mechanical properties: the acetylated form produces soft and flexible gels, while the deacetylated form produces hard and brittle gel structures. The gelation of gellan gum in solution is influenced by the addition of cross-linking agents, i.e., metal cations and glutaraldehyde. The resulting hydrogels are characterised by stability during heating, high clarity, and biocompatibility. The aforementioned pectins are anionic hydrophilic polymers (Figure 4b) containing a carboxyl group. They are built from linearly connected α-D-galacturonic acid residues and partially substituted side chains containing L-rhamnose residues or neutral sugars i.e., arabinose and galactose [60].

As early as 1996, the FDA approved the DentiPatch™ product (Noven Pharmace-uticals, Inc., Miami, FL, USA), which was the first lidocaine delivery system designed for topical anaesthesia to prevent soft tissue pain associated with dental procedures [105]. DentiPatch contains 41.6 mg of lidocaine in a 2 cm^2^ adherent karaya gum matrix covered with a polyester film and, according to the manufacturer, induces anesthesia in 2.5 min. Its safety and efficacy have been confirmed in both adults and children, although poor adhesion to the oral mucosa has been described [10].

Fernandes et al. developed oral mucosa-applied triamcinolone acetate films that are based on gellan gum and pectin. The films have high sorption capacity due to the presence of hydroxyl and carboxyl groups in both polymer molecules, with results indicating a dominant effect of gellan gum on the swelling increase of the carriers. At the same time, the films smeared after 8 h of being under in vitro conditions, which was considered satisfactory for the anticipated application and the required resistance to saliva and tongue movements, allowing prolonged contact of the drug with the application site. The prolonged diffusion of the drug has been linked to the structure of gellan gum, the molecule of which takes the form of a double helix forming a network through which the transport of the drug in the medium is restricted. The studied mucoadhesive films retained their structure during the study, showing that swelling and diffusion are the mechanisms responsible for drug release. Additionally, the interactions occurring between the particles of the hydrophilic polymers are sufficient to obtain API extended-release carriers without the use of potentially harmful cross-linking agents and organic solvents. The Korsmeyer–Peppas model was used to describe the release kinetics of triamcinolone acetate from the films, pointing to drug diffusion following Fick’s law as the mechanism for the release of the substance from the engineered films [47].

Preservation of the volume of the alveolar process after extraction is crucial to the success of the implant and prosthetic treatment. In an in vitro study, Chan et al. used gellan gum solution as a substrate to prepare 3D sponges, applied to the dental alveoli, by freeze drying. The prepared porous sponges were cross-linked by immersion in a solution of 1-ethyl-3-(3-dimethylaminopropyl)carbodiimide for 24 h and then freeze-dried again after washing. In vitro studies showed that the microstructure, porosity, and compressive modulus of sponges obtained on the basis of gellan gum at polymer concentrations of 1.5% and 1.75% were comparable to the market-leading product, i.e., Teruplug^®^ (Olympus Terumo Biomaterials corp.,Tokyo, Japan), prepared from collagen fibers with favorable hemostatic properties. The study confirmed that a 1.5% concentration of gellan gum allowed better blood adsorption properties by the carriers; in addition, the polymer itself showed an inhibitory effect on epithelial cell and fibroblast migration, which is important for preserving the alveolar shape. Based on the observed results, it was assessed that a sponge containing 1.5% polymer appears to have a high potential for use as a post-extraction dressing capable of becoming a dental filling [48].

The work by Nguyen et al. evaluated in vitro the interactions between pectin-coated liposomes and parotid gland saliva and tooth enamel to determine their potential to mimic the protective biofilm formed naturally on tooth surfaces. Liposomes were coated with different types of low-methylated, high-methoxylated, and amidated pectins (LM, HM and AM, respectively) at concentrations of 0.05% and 0.20%. Liposomes were adsorbed to hydroxyapatite and human tooth enamel using phosphate buffer and parotid gland saliva as an adsorption medium. Pectin-coated liposomes have been shown to adsorb in vitro to hydroxyapatite and act as a protective biofilm. In addition, their ability to be retained on enamel surfaces allows for their further use as protective structures for teeth, especially in patients struggling with xerostomia [106].

Gums and pectins are also frequent components of complex carriers, acting as a thickening and gelling agents, providing texture to the formulation and facilitating administration. Gracia et al. evaluated in vitro the protective effect of the semi-solid carrier TriHydra™ (GlaxoSmithKline, London, UK) containing 0.20% carboxymethylcellulose (CMC), 0.010% xanthan gum, and 0.75% copovidone on human enamel eroded with citric acid. The polymer formulation under examination was originally developed as a film-forming moisturizer in mouthwashes for the prevention and/or relief of symptoms associated with xerostomia. However, this carrier was found to exhibit effective anti-erosion activity, which is further enhanced by the addition of 300 mg/L fluoride. In the group of enamel samples in which a fluoride polymer carrier was used, reduced roughness of the surface was observed after exposure to citric acid compared to the group where water or fluoride alone was used before acid exposure. This confirms the beneficial effect of the developed solution in improving fluoride dissolution and assimilation [107].

Hamishehkar et al. studied the effect of using a triamcinolone acetonide oral paste formulation based on Plastibase^®^ containing mineral oil and polyethylene (95:5) with added polymers including pectin, gelatin, and carboxymethylcellulose sodium in the treatment of aphthous stomatitis. The selected optimized formulation containing 60% plastibase, 6.6% gelatin, 3.3% pectin, and 30% carboxymethylcellulose sodium showed the desired adhesion value, spreadability, and rheological properties after application in healthy volunteers. During a dissolution testing, a triamcinolone release profile comparable to the reference formulation Adcortyl^®^ (Bristol-Myers Squibb Pharmaceuticals, Ltd., New York, NY, USA) was obtained from the indicated formulation [108]. In conclusion, the authors indicated that the described oral paste formulation with triamcinolone acetonide showed similar properties to the reference formulation and could be used as an effective drug delivery system in the treatment of recurrent aphthous stomatitis.

### 3.2. Peptides—Collagen, Gelatin

Collagen is a natural substrate of the extracellular matrix, constituting around 30% of all human proteins, and at the same time, it is one of the main components of the connective tissue, being an adhesive that binds cells together. Moreover, it has an impact on maintaining the smoothness, elasticity, good tension, and hydration of the skin and preserving the elasticity of blood vessels, which improves blood circulation. As many as 28 types of collagen have been identified; among these, type I collagen is the best researched and most commonly used polymeric variety for obtaining materials [109]. Collagen is a polymer commonly used in dentistry, especially in dental surgery and periodontology (Table 3).

It is the basis for the production of available barrier materials (Geistlich Bio-Gide, Geistlich, Wolhusen, Switzerland), biomaterials for bone augmentation (Geistlich Bio-Oss^®^ Collagen, Geistlich, Wolhusen, Switzerland), hemostatic sponges used postoperatively, i.e., CollaPlug^®^, CollaCote^®^, and CollaTape-zimmer biomet, as well as carriers that release the substance topically i.e., PerioCol™-CG [114,115,116]. Based on collagen, resorbable barrier materials are obtained to avoid re-operation to remove non-resorbable membranes in GTR/GBR procedures. Its use in the GTR ensures hemostasis, which is a chemotactic effect against PDL fibroblasts, prevents epithelial cell migration, and enables early vessel ingrowth [117]. A meta-analysis published in 2018 and covering studies conducted between 1980 and 2014 summarized 17 randomized clinical trials and three prospective clinical studies that evaluated the efficacy of implant procedures along with alveolar bone augmentation following the use of bone substitutes and barrier collagen membranes. The study included treatments performed on 460 patients with a mean postoperative follow-up of 8.77 months. The GBR (Guided Bone Regeneration) procedure performed with resorbable collagen membranes and bone grafting materials was shown to be effective in bone regeneration, and the implants showed high survival rates [118].

Ranganathan et al. in a clinical study with 10 participating patients evaluated the effectiveness of Collaplug^®^ collagen sponges (Zimmer Biomet, Zug, Switzerland) as a post-extraction dressing after molar extraction procedures. Patients were analyzed for bone density bilaterally on radiographs, as well as clinical and subjective symptoms, i.e., swelling, pain, and wound dehiscence. Assessments were made a day, week, and 4 and 12 weeks after treatment. The results showed that patients’ wounds were healing faster after using Collaplug^®^, and bone formation was also accelerated [116]. In another study, 54 patients underwent molar extractions and compared the efficacy of using Collaplug^®^ concurrently with platelet-rich fibrin (PRF) compared to using PRF alone. The study concludes that the administration of Collaplug^®^ and PRF preserves alveolar process volume after extraction without the need for additional bone grafting procedures for future implant placement in patients [119].

Another collagen-based formulation is the PerioCol™-CG (Eucare Pharmaceuticals, Chennai, India) unilaterally rounded implant inserted into periodontal pockets, which contains approximately 2.5 mg of chlorhexidine digluconate in a biodegradable matrix of type I collagen of fish origin. During in vitro tests, the proposed carrier released approximately 40–45% of chlorhexidine within the first 24 h. The remaining dose of the substance was released linearly over 7–8 days. In clinical studies, the effects of the carrier were compared at 3 and 6 months after scaling root planing (SRP) and the simultaneous administration of the carrier into 20 periodontal pockets with a control group of patients where only SRP was applied. A statistically significant reduction in the BoP rate and improvement in the clinical location of the connective tissue attachment (CAL) were observed in the study group where a carrier was applied in addition to SRP [114].

**Figure 5:**
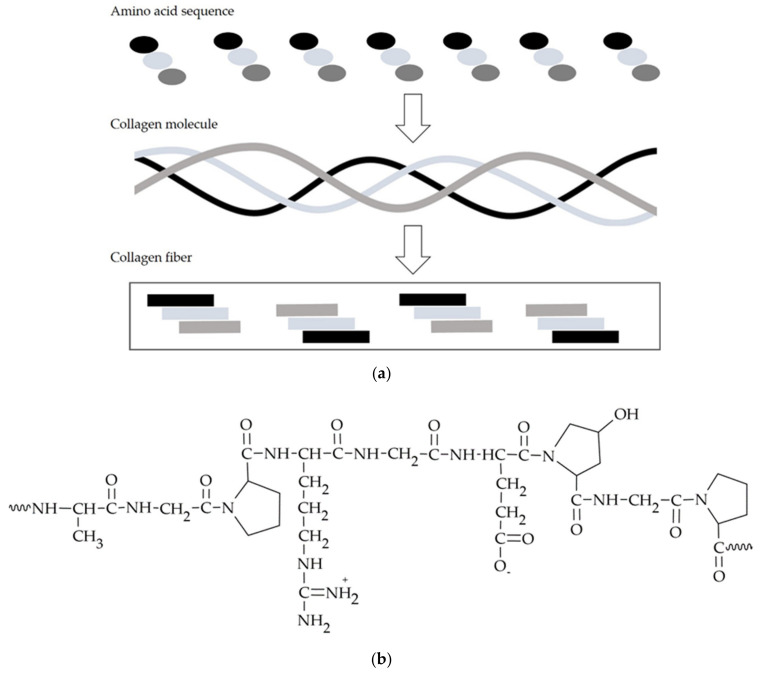
Molecular structure of collagen (**a**), gelatin (**b**) [113].

Another natural protein used in the technology of biodegradable materials and active substance carriers for dental applications is gelatin, which is obtained from the collagen discussed above by modifying the value of the protein’s isoelectric point, in either a positively charged acidic or negatively charged basic form. Changing the charge of the gelatin molecule allows both positively and negatively charged ligands to attach to it [120,121]. The presence in the gelatin molecule, shown in Figure 5b, of a double bond and functional groups such as –NH_2_, –SH, and –COOH allows its modification with other bio-molecules, thus obtaining products with different properties and applications. To improve the mechanical properties, durability, and resistance to temperature changes, gelatin is cross-linked using glutaraldehyde, formaldehyde, or genipin [122]. However, the toxicity of cross-linking compounds may limit their use and must be considered when developing a gelatin-based drug formulation [123]. An important property of gelatin is its ability to swell in cold water and, when the solution is heated to about 40 °C, to form a colloidal solution which, on cooling, forms a gel. This is due to the unfolding of the polymer’s flexible and elastic polypeptide helices at elevated temperatures and their return to a coiled and rigid structure as the temperature decreases [121]. As a result, water molecules become encapsulated and immobilized within the network’s spirally twisted amino acid chains.

As a biocompatible polymer with adhesive properties and low immunogenicity, gelatin is used in dentistry to design, in particular, drug carriers, i.e., hydrogels, porous matrices, implants, and microspheres [121,124,125]. Gelatin-based drug dosage forms have, as in the case of collagen, a high hemostatic activity, and the healing and regeneration process of damaged tissues after the use of carriers developed on their basis is significantly improved [121,122,126]. One of the most common indications for the use of a gelatin dressing in dentistry is a post-extraction wound where the alveolus fills with blood, and a clot is subsequently formed to protect the wound. In a few percent of patients, the clot is dissolved or inadvertently washed out and the extraction site is not sufficiently protected, leading to the development of a bacterial infection that manifests itself, among other things, in severe pain, and so-called alveoalgia develops within 2–3 days of the tooth extraction procedure. Applied gelatin dressings in the presence of the wound exudate swell to form a hydrogel that absorbs excess fluid while maintaining a moist environment at the site of tissue injury. Commercially available gelatin sponges, i.e., Gelfoam^®^ (Pfizer, New York, NY, USA) absorb blood in amounts many times their weight, fill the wound cavity, and thus stabilize the clot [111]. In the conducted studies, the aforementioned Gelfoam^®^ sponges were saturated with Terra-Cortril^®^ ophthalmic suspension (Pfizer Inc., New York, NY, USA) containing a mixture of antibiotic and corticosteroid (5 mg/mL oxytetracycline hydrochloride, 15 mg/mL triamcinolone acetonide) and then placed in the extraction sites of the mandibular third molars. In a clinical evaluation, the incidence of postoperative inflammatory reactions in the group treated with Terra-Cortril^®^ saturated carrier was 6.6% compared to 28.8% in untreated alveoli [127]. In another study, Rohanizadeh et al. compared the use of Gelfoam^®^ gelatin sponges with gelatin sponges prepared with hydroxyapatite and gelatin sponges, containing PLGA, as scaffolds to facilitate osteoblast penetration and settlement in tissue regeneration and recontouring. They carried out a comparative analysis of the aforementioned materials using scanning electron microscopy, radiological imaging, and evaluation of osteoblast proliferation and differentiation, including analysis of their number, replication, and differentiation rates of cells at 1 day, 3 days, and 1, 2, and 3 weeks after the appearance of osteoblast-like cells in the scaffolds. Osteoblast penetration when gelatin sponges containing hydroxyapatite were tested was similar to unmodified sponges; however, the shrinking of gelatin sponges with hydroxyapatite may counteract the positive effects of calcium on cell adhesion and proliferation. The concomitant reduction in the total surface area of sponges containing calcium ions leads adversely to less available space for migrating cells [128]. Other commercially available sponges are Gelatamp^®^ gelatin sponges used after tooth extraction, containing 5% colloidal silver (Gelatamp gelatin sponge with colloidal silver, Coltène/Whaledent Ltd., Burgess Hill, UK), from which Ag^+^ ions, which are highly reactive against bacteria such as *Escherichia coli* and *Staphylococcus aureus*, are released in a prolonged way under moist wound conditions. Gelatamp^®^ has both hemostatic and bactericidal effects, remains in the alveolus, and completely resorbs within 4 weeks. The efficacy of Gelatamp^®^ in preventing alveolar desiccation after the removal of mandibular retained teeth was evaluated in a group of 976 patients who had 1350 teeth removed, observing complications in only 0.44% of those treated, compared to 4.44% recorded in a control group in which the material was not used [112]. The antimicrobial effect of silver ions was confirmed during in vitro studies on MRSA-infected rat cranial bones, where statistically better healing effects were observed after treatment with Gelfoam^®^ (Gelatin, Pfizer Inc., New York, NY, USA) [129]. Clinical evaluation of the use of Gelatamp^®^ to avoid postoperative alveolar dryness and bleeding was also conducted in patients receiving anticoagulants, confirming the effectiveness of using the sponge as a topical hemostatic agent in this group of patients, without the need of discontinuing the administration of the anticoagulant before extraction [130].

One of the most well-known preparations derived from hydrolyzed gelatin cross-linked with glutaraldehyde is Periochip^®^, which is used intranasally in treating patients with periodontitis (Perio Products Ltd., Jerusalem, Israel) [110]. Periochip^®^ takes the form of an orange-brown, unilaterally rounded leaf, measuring 5 mm × 4 mm × 0.3 mm and weighing 7.4 mg. The leaf contains 2.5 mg of chlorhexidine digluconate incorporated in a biodegradable matrix, which is gradually hydrolyzed after application; at the same time, it releases the substance until it is completely degraded. A randomized study involving 15 patients compared the efficacy of using Periochip^®^ together with a scaling root planing (SRP) procedure at 1 and 3 months after application compared to using an SRP procedure alone [131]. After implantation of the carrier into 30 periodontal pockets 5 to 7 mm deep, clinical indicators were evaluated, and a bacteriological examination of the gingival pocket swab was performed. In the study group, there was a statistically significant reduction in pocket depth from 1.97 to 1.07 mm and a concomitant reduction in the number of periopathogens present [131]. During an in vitro dissolution testing, it was observed that the release of chlorhexidine from the Periochip^®^ carrier occurred in a biphasic manner [131], with 40% of the substance released during the first 24 h and the remaining 60% of chlorhexidine released over a further 7–10 days. The resulting release profile is the result of an initial rapid release and diffusion of chlorhexidine from the implant, and in the second stage, it is the result of the subsequent enzymatic degradation of the carrier [132].

Pakzad et al. obtained a thermosensitive extended-release gel of metronidazole targeting pocket application for the treatment of periodontitis. The gel was based on chitosan, gelatin, and glycerol phosphate. The key properties of the obtained carrier, i.e., gelation time and temperature, were modified by selecting the concentration of gelatin and glycerolphosphate, while the gelation efficiency (gel strength) increased significantly with increasing gelatin content in the formulation. In an in vitro evaluation of *Clostridium sporogenes* cell cultures, cell growth inhibition and the biocompatibility of the developed material were confirmed. As a result of the controlled gelation process, the invasive effect of the hydrogel on tissues after application to the periodontal pocket was minimized, and the application properties of the carrier, which thickened under physiological conditions at body temperature, were improved [35].

### 3.3. Semi-Synthetic Polysaccharides—Cellulose Derivatives

**Figure 6:**
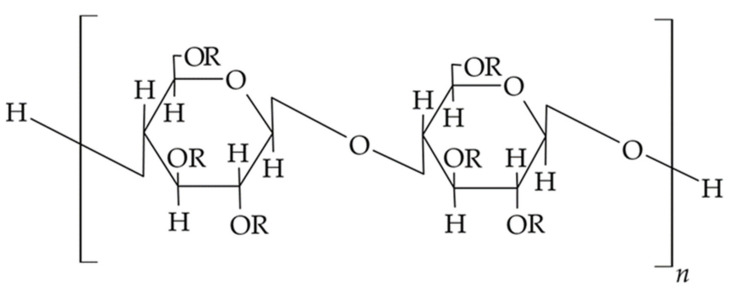
Molecular structure of cellulose ether [133].

A group of excipients used in carrier technology for topical oral apposition consists of cellulose ethers, which include methylcellulose (MC), ethylcellulose (EC), hydroxyethylcellulose (HEC), hydroxypropyl cellulose (HPC), carboxymethyl cellulose (CMC) and its sodium salt (CMCNa), and mixtures of ethers: hydroxypropyl methylcellulose (HPMC) and hydroxyethyl methylcellulose (HEMC), see Figure 6 and Table 4.

Depending on the molar mass, the amount of reactants used to obtain cellulose ethers—i.e., alkyl or aryl halides, alkene oxides, unsaturated compounds with a free electron group, and the degree of saturation of the alcohol groups of the polymer, the resulting derivatives differ in their physicochemical properties and solubility [134]. For example, as a result of introducing hydrophobic alkyl groups to the side chains of a cellulose molecule, the polymer additionally gains the ability to form thermosensitive gels, which are used in applications. The longer the attached alkyl group, the faster the gelation process of the modified polymer [135]. The choice of type and chemical structure of polymer introduced to the pharmaceutical composition—a cellulose derivative or a mixture of polymers—significantly influences the physicochemical properties of the developed carriers, i.e., its mucoadhesion strength, dissolution time, mechanical strength, flexibility, ability to incorporate the active substance, and as a result, application properties. In the case of hydroxypropyl methylcellulose, due to the different content of methyl and hydroxypropyl groups in the polymer molecule, it is possible to obtain aqueous dispersions with different properties. Polymers with a high content of hydroxypropyl groups relative to methyl groups, in the presence of saliva, swell to form a dense film, delaying the diffusion of the active substance and thus prolonging its release time. On the other hand, HPMC substitution types with lower contents of hydroxypropyl groups are characterized by high viscosity, mucoadhesion, stiffness, and mechanical resistance. Hence, mixtures of polymers with different degrees of substitution or their combination with other polymers, i.e., polyvinylpyrrolidone, polyvinyl alcohol, carbomer, gelatin, or chitosan, are often used to obtain a carrier with optimal properties [134,136].

The cellulose derivatives carboxymethyl cellulose sodium and hydroxyethyl cellulose, combined with gelatin, were used to develop porous matrices with metronidazole administered topically into the periodontal pocket (Table 5). The mechanical properties of the carriers depended on the amount of gelatin introduced. The gelatin provides a rigid backbone for swelling elastic cellulose derivatives. It has been shown that the covalent bonds formed between amino acids in the gelatin molecule and the formed dense pore network maintain the structure of the carrier after administration into the pocket and facilitate its application. Hydrophilic cellulose derivatives swelled in volume and the matrix filled the periodontal pocket while gradually releasing the drug. In vitro studies have confirmed the high antimicrobial efficacy of the carrier against osteoblast and fibroblast lines and moderate cytotoxicity of the developed matrices against these cell lines. After clinical application of hydroxyethylcellulose dressings in a group of 23 patients with stage 2 or 3 periodontitis, a favorable improvement in clinical parameters, including a decrease in periodontal pocket depth and bleeding, were observed 30 days after application [137].

Mucoadhesive films as drug carriers may have better application properties in terms of the flexibility and convenience of administration compared to tablets. It is emphasized that films act as a protective dressing, influencing the reduction of pain, which effectively supports the treatment of the disease. Additionally, in combination with mucoadhesive gels, films can remain on the mucosa for a longer period of time, which is also important when the formulation is applied to lesions located on the oral mucosa. Laffleur et al. [136] used various semi-synthetic cellulose derivatives, i.e., ethylcellulose, hydroxyethylcellulose, hydroxypropyl methylcellulose, and carboxymethyl cellulose sodium salt with polyvinylpyrrolidone K30 or K90 and triethyl citrate as a plasticizer. Furthermore, they designed mucosal films with the above-mentioned derivatives for the targeted application of allantoin to the mucosa in patients suffering from dry mouth. Evaluation of the physicochemical properties of the formulations, i.e., buccal adhesion and degree of swelling, indicated differences depending on the composition of the cellulose derivatives used. Based on in vitro and ex vivo evaluation, the authors concluded that the selected film formulations, due to the moisturizing nature of the allantoin–cellulose derivative combination, are promising formulations that, when applied to the patient, have the potential to reduce friction between the mucosa and the teeth and food consumed in dry mouth therapy.

In the study by Ammar et al. [139], HPMC- or EC-based mucoadhesive films with the addition of CMC sodium, chitosan, or Carbopol were designed. Films served as carriers for fluticasone propionate (2%) in the treatment of lichen planus-like or other erosive lesions located on the oral mucosa. Of the 14 formulations developed, two containing, respectively, 65% HPMC, 25% EC, 10% CMC, and 65% HPMC, 25% EC, 10% Carbopol and, in each case, the addition of propylene glycol as a plasticizer were evaluated in clinical trials. The adhesion of the carriers to the mucosa over a period of 4 to 6 h was confirmed, and at the same time, no adverse effects after application were observed. The results of studies in healthy volunteers showed that the formulation containing HPMC, EC, and CMC, when applied, allowed a controlled release of fluticasone propionate over a period of 10 h, which is considered a favorable value for the topical treatment of oral mucosal diseases.

Yamamura et al. [146] used a three-layer HPC adhesion film containing dibucaine (0.25 mg/cm^2^) to treat oral ulcers caused by chemotherapy or radiotherapy. The film consisted of a non-adhesive layer, a substance-containing intermediate layer, and an adhesive layer, with the addition of ethylcellulose to the non-adhesive layer preventing the film from sticking during application alongside the addition of pectin to the adhesive layer of the film, improving its adhesion to the mucus. In a pilot study with patients, the three-layer dibucaine film was easy to apply, well-tolerated, and effective in relieving pain in oral ulcers, which may ultimately improve the quality of life of patients with eating disorders.

Oguchi et al. [147] used a mucosal HPC film containing tetracaine, in combination with ofloxacin, miconazole nitrate, guaiazulene, and triacetin as a plasticizer, for the treatment of radiation-induced acute oral mucositis. The film was applied to 25 patients with radiation-induced acute oral mucositis. Oguchi et al. compared the severity of oral pain in the carrier-applied group with non-carrier-applied patients. They observed a significant reduction in the mean duration of pain after application. No acute or chronic side effects of the film were reported during the 3-year follow-up period. The developed polymer film, containing local anesthetics and antibiotics, has proven useful in relieving pain associated with radiation-induced acute oral mucositis, preventing secondary oral infections without causing adverse effects.

Repka et al., basing on HPC in combination with hydroxypropylmethylcellulose (HPMC), have obtained a lidocaine film by means of hot extrusion. The film had adhesion properties to the intestinal mucosa of a rabbit and prolonged lidocaine release during in vitro studies [149]. During dissolution testing of the substance, an initial period of increased release, beneficial for a rapid onset of action, was followed by a prolonged release of the drug relevant for potential analgesic use in dental procedures.

In the study by Kohda et al. [150], polymeric films with 30% lidocaine content prepared on the basis of EC and HPC in a 1:1 ratio showed significant adhesion to the buccal mucosa for about 60–120 min in clinical evaluation. The release of lidocaine from the drug reservoir film was controlled in vitro, which was explained by an initial low release of HPC with the substance, followed by a decrease in the release rate due to swelling of the film as a result of fluid penetration and release of the substance as a result of diffusion from the swollen HPC matrix. Signs of anesthesia were observed in all volunteers of the clinical trials. They were a pharmacological effect of lidocaine, which persisted throughout the adhesion of the carrier to the buccal mucosa.

In a study by Mohammed et al. [143], mucosal tablets containing 20 mg of miconazole and releasing the substance in a controlled manner were obtained using hydroxypropyl methylcellulose, carboxymethyl cellulose sodium salt, Carbopol 934P, and sodium alginate. Depending on the formulation, the tablets persisted on the oral mucosa for 2.45 to 3.65 h after application. No adverse effects were observed in patients using the drug. Compared to the authorized miconazole product available as an oral gel, the tested mucoadhesive tablets released the drug in a prolonged manner, without the presence of the initial peak characteristic of release from unmodified dosage forms. After application of the tablets to the mucosa, miconazole was determined to be present in the saliva of patients for more than 8 h, which had a beneficial effect on the therapy of oral mycosis, reducing the duration of therapy and hindering the emergence of resistant strains.

Ceschel et al. [142] used different polymers, i.e., HPMC, Carbopol 974P, or polycarbophil, to produce mucoadhesive tablets containing 1 mg of hydrocortisone acetate for the treatment of oral mucosal lesions. Tablets containing HPMC compared to other mucoadhesive polymers during in vitro studies did not show a favorable release profile. A significant proportion of the drug was released within the first hour of the study, which did not indicate prolonged drug release. Healthy volunteers participating in a clinical trial evaluating the proposed hydrocortisone tablets did not report adverse reactions associated with the application of this formulation. Depending on the HPMC concentration used in the formulation, the tablets persisted in the mouth from 25 min to over 2.25 h. Unfortunately, in vivo studies did not determine at what rate and for how long hydrocortisone was released in the oral cavity.

The mucoadhesive tablets developed by Ceschel et al., depending on the percentage of hydroxypropyl methylcellulose in the formulation, showed different degrees of release and diffusion capacity of chlorhexidine across the mucosa in vitro. As the HPMC concentration in the tablet increased, chlorhexidine was released in a prolonged, near-controlled manner. At the lowest HPMC concentration used, the diffusion of chlorhexidine through the mucosa was the lowest [151].

Kamel et al. prepared bioadhesive tablets obtained by pressing a mixture of HPC and carbomer. The tablets adhered to the gingiva and underwent gradual erosion over a period of 8 h, providing a prolonged effect of the citrus oil incorporated in them, which was used in the treatment of aphthae [135].

Fini et al. [152] used cellulose derivatives during in vitro studies to design carriers for chlorhexidine, which is a frequently used antiseptic in the treatment of gingivitis and periodontitis. The developed mucoadhesive gels based on cellulose derivatives—CMC, HPC, and HPMC—showed the ability to interact with mucin, which is a component of mucus covering the buccal epithelium, thus allowing obtaining a prolonged residence time of the carriers on the mucous membrane. The use of CMC in gels has allowed the rapid release of the active ingredient from the carrier, which may find application in clinical acute conditions where rapid release and high concentration of the anti-inflammatory agent at the same time is intended. In contrast, obtaining a gel with CMC (2%) and HPC (3%) resulted in a prolonged and controlled release of chlorhexidine over 4 h [152]. The study by Bansal et al. analyzed the dissolution testing of CMCNa-based mucosal gel satranidazole used for the treatment of periodontitis. The release of the drug from the carrier was favorably prolonged and controlled for 8 h, all according to Fick’s diffusion law. Moreover, the rate of drug transport from the hydrogel matrix to the surrounding medium diminished with decreasing drug concentration in the carrier, corresponding to the described process, which was based on Higuchi’s model [145].

### 3.4. Biodegradable Synthetic Polymers

#### 3.4.1. Polyvinyl Alcohol (PVA)

**Figure 7:**
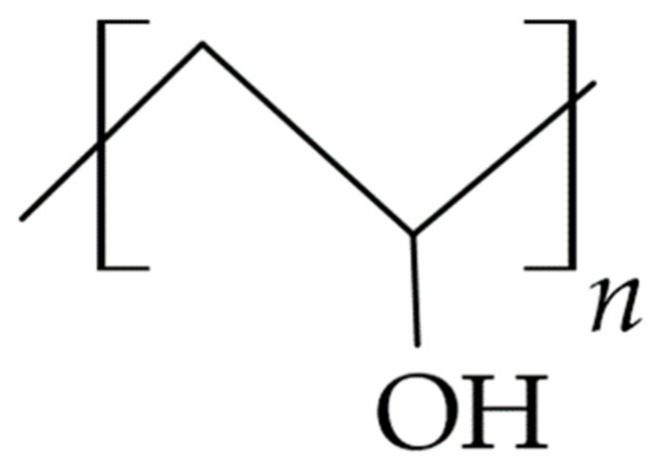
Molecular structure of PVA [154].

PVA (Figure 7) is one of the most widely used synthetic polymers for design of carriers applied topically to the oral mucosa [153]. It is a non-ionic polymer that dissolves in aqueous solutions, increasing their viscosity, with low molecular weight PVA varieties yielding lower viscosity formulations. Polyvinyl alcohol is a macromolecular compound characterized by a high hydrogen bonding capacity, which has been shown to be a prerequisite for the occurrence of mucoadhesion. The study confirmed that the bioadhesion strength of PVA rises with the increase in polymer molar mass (>100,000), polymer chain length, and degree of elasticity, which are properties further determining their diffusion into the mucin layer and entanglement with mucus components.

PVA-only hydrogels produce stiff films with low elasticity after solvent evaporation, limiting the independent use of the polymer as a carrier component. Gajra et al. [155] designed a PVA-based mucoadhesive hydrogel film for the administration of econazole for the treatment of oral candidiasis. A mucoadhesive hydrogel film containing 15% polyvinyl alcohol, after seven freeze–thaw cycles conducted to increase the degree of cross-linking, showed a prolonged release of econazole, lasting over 12 with similarity to zero-order kinetics. During in vitro tests using the buccal mucosa of a goat, the authors confirmed that the obtained carrier has good mucoadhesive properties, which is important to obtain the in vivo prolonged contact time of the drug with the buccal mucosa. It is also important for the efficacy of oral candidiasis treatment, especially that the drug released from the optimized film can inhibit the growth of *Candida albicans* for more than 12 h, resulting in better compliance and higher therapeutic efficacy.

By combining PVA with other polymers, i.e., alginates, chitosan, cellulose derivatives, and PVP, it is possible to obtain biocompatible, biodegradable films with optimal properties and low toxicity [156], which at the same time have good durability, smear resistance, and thermal stability [157]. Nafee et al. [158] on the basis of PVP and selected polymers, i.e., carboxymethylcellulose sodium salt, chitosan, polyvinyl alcohol, hydroxyethylcellulose, and hydroxypropylmethylcellulose, received carriers in the form of a 10 mm diameter mucosal patch containing 10 mg of miconazole nitrate for use in the treatment of patients with oral candidiasis. In a study to evaluate bioadhesion, elasticity, swelling, and release in vitro, a patch based on 10% PVA and 5% PVP, with a prolonged release of miconazole over 5 h, was selected for further in vivo evaluation. An in vivo patch study in healthy volunteers confirmed constant drug levels in saliva, guaranteeing the comfort of use for at least 6 h (C_max_ 120.275 μg/mL, T_max_ 2.3 h, AUC_0–6_ 370. 29 h) in comparison to the commercial oral Daktarin^®^ oral gel product (Janssen-Cilag International, Beerse, Belgium), which achieved a high release of miconazole from the carrier (C_max_ 549.897 μg/mL, T_max_ 0.08 h, AUC_0–6_ 182.12 h), rapidly decreasing after the first hour of use. For both tested formulations, the T > MIC value was also determined. It indicates the time at which the last measured drug concentration in saliva is above a certain value of MIC. For miconazole nitrate against *Candida albicans*, the value is 5 μg/mL. The recorded T> MIC values for the tested miconazole mucoadhesive patch and Daktarin^®^ oral gel were 6.1 h and 1.313 h, respectively. The authors indicated that the mucoadhesive patch had a greater ability to maintain elevated salivary concentrations of the therapeutic substance, despite the administration of a lower drug dose of 10 mg compared to the gel where 25 mg was used.

Padula et al. [159] developed lamination-generated films with PVA, sorbitol as a plasticizer, and without or with the addition of an acrylic polymer (Plastoid^®^ E35H, Gaggiano, Italy) for the buccal delivery of lidocaine, in order to diminish the pain sensation caused by needle pricks during local anesthesia in dentistry. Combining PVA with an acrylic polymer improved lidocaine transport across the esophageal epithelium of a pig used as a model of the non-keratinized buccal mucosa. The results obtained in this study confirm the validity of using a mucoadhesive film with an anesthetic agent for application to the mucosa. This avoids the disadvantages of semi-solid preparations, such as short residence time, while ensuring the adequate release of the drug into the mucosa.

#### 3.4.2. Polylactides (PLGA, PLA)

**Figure 8:**
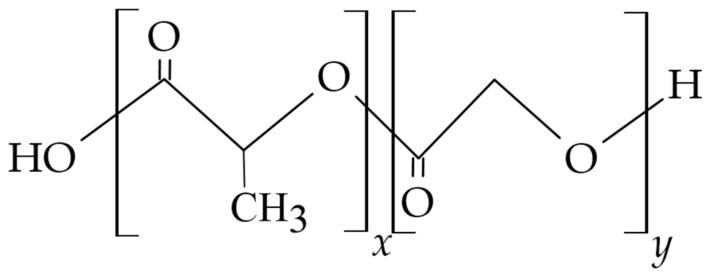
Molecular structure of PLGA [160]. *x*: units of lactic acid, *y*: units of glycolic acid.

Poly(lactic-co-glycolic acid) (PLGA, Figure 8) is a biodegradable and biocompatible copolymer of DL-polylactic acid (PLA) and poly(glycolic acid) (PGA) is classified as a thermoplastic aliphatic polyester, where the polymer structure can be formed by molecules of virtually any molecular weight. By changing the molecular weight of the polymer and the ratio of lactide to glycolide monomers in the polymer chain, it is possible to obtain the type of polymer with different degrees of crystallinity, polydispersity index, mechanical strength, swelling capacity, hydrolysis rate, and degradation time. PLGA copolymers with high PLA content show higher hydrophobicity, thus absorbing less water and degrading more slowly due to the presence of methyl side groups in PLA monomers. The higher crystalline PGA content in PLGA leads to a lower degree of crystallinity of the copolymer and a higher degree of hydration and faster hydrolysis. PLGA can be processed into any structure and form of different sizes, and the polymer is characterized by high chain rigidity. However, in the body, it gradually biodegrades to lactic and glycolic acid by hydrolysis of ester bonds, which is advantageous for developing absorbable, implantable drug carriers. Studies show that the rate of release of substances from PLGA-based polymeric matrices is affected by storage conditions and the type of drug incorporated, in addition to the changes in the surface and shape of the carrier as a result of biodegradation [160].

Given these properties of the polymer, it is often considered as a reference polymer when developing new nanoparticles and microparticles (Table 6) with both hydrophobic and hydrophilic substances incorporated in them. Drug carriers and PLGA-derived materials further enhance cell proliferation and adhesion to their surface, which is used in tissue engineering.

Gad et al. [161] studied in situ derived implants based on PLGA or PLA polymers dissolved in N-methyl-2-pyrrolidone for topical application of secnidazole and/or doxycycline to periodontal pockets of patients with periodontitis. In ongoing studies, carriers obtained in situ with two polymer percentages, 25% and 35% respectively, and their combination with secnidazole, doxycycline, or a mixture of both antibiotics were evaluated. An increase in polymer concentration in each case resulted in an increase in viscosity and a decrease in the sum of the percentage of active substance released from the carrier after 24 h of testing, with PLA-based implants showing slower drug release rates compared to PLGA-based implants. In an in vitro study, it was found that of the formulations tested, an implant containing 25% PLGA with secnidazole and doxycycline hydrochloride released antibiotics most quickly and showed greater antibacterial efficacy against aerobic and anaerobic bacteria compared to the doxycycline-containing product Atridox^®^ (CollaGenx Pharmaceuticals, Inc., Newtown, CT, USA) for the treatment of periodontitis.

An in vitro study conducted by Torshabi et al. [162] evaluated the properties of PLGA-based formulated microspheres with three incorporated antibiotics: minocycline, metronidazole, and ciprofloxacin. The antibiotic release rate and antibacterial efficacy of the developed carrier against *A. actinomycetemcomitans* periopathogens were analyzed. In vitro release of the antibiotics from the microspheres occurred in a prolonged manner over a period of 16–18 days, with antibacterial activity exhibited by inhibiting the proliferation of *A. actinomycetemcomitans* over a period of 11 days. The study highlights that these microspheres can be an effective alternative to systemic antibiotic therapy for the treatment of periodontal infections, providing local, controlled, and prolonged drug release.

Yue et al. developed a 4 mm × 5 mm × 0.5 mm pad-like carrier of compressed polymer microspheres derived from PLGA with incorporated chlorhexidine as free base or chlorhexidine digluconate in association or inclusion complexes with methylated beta-cyclodextrin (MBCD) or hydroxypropyl-beta-cyclodextrin (HPBCD) for topical subgingival administration in the treatment of periodontitis. In a preliminary assessment, complexation of the poorly water-soluble chlorhexidine with HPBCD resulted in a 62% higher encapsulation efficiency of the substance and a prolonged release time of up to 2 weeks compared to its complexation with the more lipophilic MBCD. In contrast, complexing the water-soluble chlorhexidine derivative with MBCD improved encapsulation efficiency by 12% and prolonged its release compared to both chlorhexidine digluconate alone and its complex with HPBCD. Subsequent studies have shown that chlorhexidine released from PLGA-based pads containing inclusion complexes of the substance is active against *P. gingivalis* and *Bacteroides forsythus* in an agar plate test. During dissolution testing, it was shown that the release of chlorhexidine and its derivatives from degradable polymeric matrices could be modulated by their complexation with cyclodextrins, and the developed carrier shows a prolonged release of the substance for at least 2 weeks and may be suitable for the topical delivery of antibacterial and anti-inflammatory substances in the treatment of periodontitis [163].

Nanoparticles < 220 nm in diameter and positively or negatively charged based on PLGA and Pluronic F-108 were also used in the study of Klepac-Ceraj et al. to incorporate methylene blue (10%) as a photosensitizer for potential dental applications in photodynamic therapy (PDT) of periodontitis. To modify the surface properties of the nanoparticles and give them a positive or negative charge, the researchers respectively used cetyltrimethylammonium bromide (CTAB) or a 10% addition of the tri-block copolymer Pluronic F-108^®^ as a surfactant. For biological evaluation of the nanocarriers, samples of the bacterial suspension were treated for 20 min with photosensitizer incorporated in the nanoparticles and irradiated for 5 min with red light at 665 nm. In an in vitro evaluation, it was confirmed that positively charged microspheres with methylene blue showed greater phototoxicity against both the planktonic phase, which was cultured from swabs taken from patients with periodontitis, and the biofilm compared to anionic nanoparticles with incorporated methylene blue and the action of methylene blue alone. The results suggest that cationic PLGA nanoparticles have the potential to be used as photosensitizer carriers in oral photodynamic therapy. Still, the effect of the applied concentration of methylene blue on the control of biofilm organisms requires additional studies of the nanoparticles’ physical properties and the determination of the optimal PDT parameters for the effective elimination of different biofilm bacterial species [164].

Nanoparticles containing minocycline were also designed based on PLGA, and the efficacy of the carrier after its subgingival topical application was evaluated by observing changes in ongoing inflammation. The available therapeutic product Arestin^®^ (OraPharma, Inc., North Bridgewater, NJ, USA), containing minocycline hydrochloride (1 mg) incorporated in nanoparticles with PGLA, released the antibiotic in a prolonged manner for 2 weeks in clinical trials, allowing local drug concentrations > =300 μg/mL in gingival crevicular fluid [165]. Product studies have confirmed the efficacy of intracarotid carrier administration in reducing gingival inflammatory indices and reducing periodontal pocket depths [165,166]. Another clinically evaluated product for the subgingival application is Atridox^®^ (CollaGenx Pharmaceuticals, Inc., Newtown, CT, USA), which releases doxycycline over 7 days. The product is supplied in two syringes A and B, the contents of which should be mixed before use. Syringe A contains 450 mg of a gel based on bioabsorbable PLA dissolved in 63.3% N-methyl-2-pyrrolidone (NMP), while syringe B contains 50 mg of doxycycline hyclate in substantia, which is equivalent to 42.5 mg doxycycline. In the patient group in which the product was used in addition to the standard scaling and root planing (SRP) procedure, compared to the control group, a reduction in pocket depth (PD) and clinical attachment level (CAL) was observed. Three- to six-month follow-up periods in studies involving larger numbers of patients (45–60 patients) confirmed that statistically significant improvements in the assessed clinical parameters were achieved following the use of a doxycycline product in patients after SRP compared to SRP alone [167,168].

The PLGA copolymer was also used to develop a barrier membrane for Guided Bone Regeneration (GBR) in patients with periodontitis. The aim of the study by Saarani et al. was to evaluate the antimicrobial efficacy of an applied trilayer membrane composed of PLGA, nano-apatite (NAp), and lauric acid (LA) in a disk shape of 6 mm diameter against Gram-negative bacteria of the genus *Fusobacterium nucleatum* and *P. gingivalis* in a disk diffusion method. The developed membranes containing 2% and 3% LA showed bacteriostatic and bactericidal activity against both mentioned bacterial strains, while increasing the LA concentration in the carrier guaranteed prolonged bactericidal activity of the membrane. A study by Saarani et al. showed that the incorporation of LA into the GBR membrane slowed the growth and proliferation of Gram-negative bacteria, which is beneficial in the treatment of periodontal disease by preventing the inflammatory response and promoting bone tissue regeneration [169].

#### 3.4.3. Polycaprolactone (PCL)

**Figure 9:**
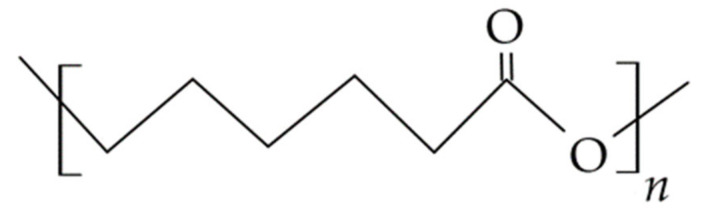
Molecular structure of PCL [160].

Polycaprolactone (PCL, Figure 9) is a biodegradable, biocompatible, semi-crystalline polyester obtained by ring-opening polymerization of the monomer ε-caprolactone. The polymer is easily recyclable, additionally miscible, and compatible with other polymers, increasing the biodegradability of carriers and biomaterials obtained with its participation. As a result of copolymerization of PCL with hydrophilic monomer i.e., PEG, in comparison with homopolymer, water penetration is increased, and the degradation time of materials obtained on their basis is shorter; at the same time, their biological compatibility is higher.

Due to its slow degradation, lack of toxicity, and ease of diffusion of the incorporated drugs, the polymer is used to develop implantable carriers/implants with sustained release according to zero-order kinetics. PCL-based materials are also used as scaffolds in tissue engineering and as micro and nanocarrier matrices for topically applied drugs in dentistry, stimulating tissue regeneration and inducing the differentiation of dental pulp cells [160,170]. Nasajpour et al. [171] developed and characterized a flexible, biodegradable, and easily implantable poly(caprolactone) (PCL) membrane containing zinc oxide (ZnO) nanoparticles for GTR, which reduces the development of bacterial infection and promotes alveolar bone regeneration. Membranes were produced using electrospinning technology of a 10% PCL solution in hexafluoroisopropanol containing 0.5 or 1% ZnO nanoparticles. The addition of ZnO was expected to provide not only antimicrobial activity but also to improve the osteoconduction of the barrier membrane. The functionality of the membrane was evaluated 6 weeks after application in an animal model, after generating an alveolar bone defect in rats and applying the membrane. A greater increase in bone tissue was observed in rats with the barrier membrane compared to animals without it. The results confirmed that the modified PCL membrane containing 0.5% ZnO nanoparticles exhibited osteoconductive and antimicrobial properties, without any negative effect of nanoparticle addition on biocompatibility, indicating its potential as a material for periodontal tissue engineering.

Shim et al. [172] compared barrier membranes obtained using extrusion-based 3D printer incremental technology based on polycaprolactone (PCL) and polycaprolactone with β-tricalcium phosphate (PCL/β-TCP) with a commercially available collagen membrane in terms of efficacy in Guided Bone Regeneration (GBR) procedures. In this study, β-TCP was used to increase the degradation rate of PCL membranes, as β-TCP has been shown to biodegrade in vivo within 3–6 months. In contrast, PCL biodegradation takes more than 12 months, and scaffolds should degrade slowly enough to conserve space during the initial growth of new bone but fast enough to provide room for new bone formation. In vitro studies showed that PCL/β-TCP-based membranes had superior physical, biological, and mechanical properties compared to membranes derived from PCL alone. In vivo evaluation in three beagle dogs with alveolar bone defects showed that the defects were covered with different membranes, and CT scans and histological analyses 8 weeks after surgery showed that 3D-printed PCL/β-TCP membranes were more effective than those made from PCL alone. The 3D-printed PCL/β-TCP membrane was more effective in Guided Bone Regeneration compared to the collagen membrane used for buccal bone defects, indicating that the PCL/β-TCP membrane could potentially be used as a resorbable GBR membrane for the treatment of alveolar bone defects. Furthermore, the observed higher structural stability of the 3D-printed PCL/β-TCP membrane suggests that it is an alternative to collagen membranes for GBR.

Xue et al. [173] electrospun PCL and gelatin to obtain metronidazole microfibers with a diameter of 400 nm, forming a membrane for GTR GBR. The microfibers were loaded with 20 wt % halloysite clay nanotubes, 50 nm in diameter and 600 nm in length, in which metronidazole was also incorporated. Due to the membrane structure formed by nanotubes incorporated within the electrospun fibers, an enhancement in the tensile strength of the membrane was achieved, and in vitro studies reported a prolonged release of metronidazole for 20 days, compared to a 4-day release of the substance obtained from microfibers alone. Prolonged release of metronidazole prevented the colonization of the membranes by Gram-negative *Fusobacterium* anaerobes for a period of 3 weeks, while eukaryotic cells could continue to adhere to and multiply on the drug-containing membranes. This indicates the potential of complex electrospun membranes for clinical applications in periodontitis therapy.

#### 3.4.4. Poloxamers

**Figure 10:**
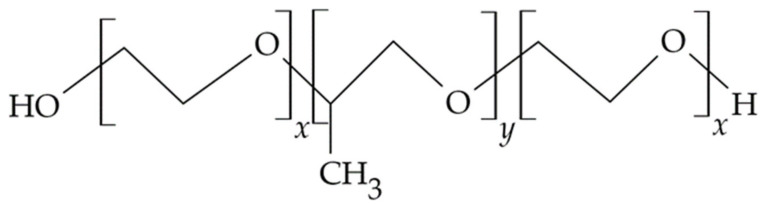
Poloxamer molecular structure [174]. *x*—hydrophilic polyethylene glycol block, *y*—hydrophobic block of polypropylene glycol.

Poloxamers/Pluronics (Figure 10) are non-ionic, amphiphilic triblock copolymers whose single molecule consists of centrally located blocks of poly(propylene oxide) surrounded by poly(ethylene oxide) chains facing the aqueous phase. In an aqueous solution, monomers self-organize into micelles, improving the availability of encapsulated drugs and acting as carriers for poorly soluble substances with variable pharmacokinetics and low stability in the physiological environment. By changing the size of individual blocks and altering the compound’s molecular weight, poloxamers with different mechanical properties, microstructure, wettability, or mimicking the behavior of different tissue types are obtained. Poloxamer-based drug dosage forms are biocompatible, erodible, and biodegradable through a gradual dissolution of the matrix to lower molecular weight fragments. Due to additional properties of the polymer, i.e., thermosensitivity or low immunogenicity, in the drug formulation, technology poloxamers are used mainly to obtain semi-solid topically applied drug carriers, i.e., gels formed in situ [174,175].

Aithal et al. [176] for use in the treatment of periodontitis proposed a bioabsorbable nanoemulsion obtained by the spontaneous emulsification based on cinnamon oil as the oil phase, Tween 80, and Carbitol^®^ as the surfactant-cosurfactant mixture (S_mix_) and a 23% solution of Poloxamer 407 as the dispersing phase, in which quercetin, a substance with antimicrobial and anti-inflammatory properties, was dissolved. In the evaluation of the nanoemulgel formulation, a predetermined concentration of quercetin was used to ensure a minimum colony inhibitory concentration (MIC) of the periopathogens *P. gingivalis* and *T. forsythia* in the range 75–125 µg/mL. During quercetin dissolution testing, 92.4% quercetin was released from the nanoemulsion after 6 h of testing, compared with <3% released under the same conditions from a gel formulation containing equivalent quercetin content. In addition, the conducted studies confirmed the presence of a sol–gel phase transition of the formulation as a function of temperature, thus proving that nanoemulgel with quercetin can be successfully used in a low-viscosity formulation that is easy to apply with a syringe, transforming into an in situ viscous gel at body temperature, which is extremely beneficial from the point of view of the design and development of delivery systems for periodontal inflammatory diseases.

In a clinical study conducted by Gad et al. [177], patients with periodontitis were treated with a poloxamer gel containing a formulation of solid lipid microparticles (SLM) optimized in preliminary studies containing doxycycline hydrochloride and metronidazole in molecular dispersion and obtained based on different types of lipids: glyceryl behenate or tribehenin, purified phospholipid, tripalmitin, stearic acid, and different concentrations and types of surfactants. In the study, the microbiological and clinical evaluation of selected SLMs in patients with periodontal disease was performed sequentially, with patients divided into three groups and a scaling and root planing (SRP) procedure performed in all of them. The formulated gel, with solid lipid microparticles with doxycycline and metronidazole, after application into the periodontal pockets, showed a statistically significant reduction in the number of periopathogenic bacteria in the periodontal pockets compared to the scaling and root planing (SRP) procedure applied in the control group or to the gel containing doxycycline and metronidazole administered in the second control group. The gel in the second control group was based on poloxamer P407 (13% above mentioned), poloxamer P188 (11% above mentioned), and hydroxypropyl methylcellulose (HPMC) as a mucosal component (0.1% above mentioned). An in vivo study confirmed the efficacy and safety of the SLM gel in the treatment of periodontitis, indicating that the developed gel offers a suitable dosage form that can be injected directly into the periodontal pocket as an adjunctive treatment to the SRP procedure. The researchers explain the effectiveness of the formulation by the high ability of SLM to physically bind to tissues, which reduces its elution from pockets by gingival fluid.

Abdel-Hamid et al. proposed the use of mebeverine hydrochloride, which has a diastolic effect, in hydrogels based on 20% Poloxamer-407 with 0.5% hydroxypropyl cellulose and excipients, including a preservative and flavor enhancer, for the treatment of painful oral conditions, i.e., Wilson’s lichen, recurrent aphthous, erythema multiforme, and Behçet’s syndrome. A clinical evaluation of pain relief efficacy, in a randomized single-blind, split-mouth-design study, showed a greater anesthetic effect of the proposed hydrogel compared to the commercially available formulation Lidocaine HCl gel^®^, as well as better healing of buccal mucosal wounds after application of the formulation [178].

**Table 6:** Biodegradable synthetic polymers used in topically applied carriers in dentistry.

Polymer	Carrier	Drug
PVA	film	ornidazole [179] econazole [155] pvpi [180] lodocaine [159]
Polylactides	fibers	lidocaine and epinephrine [117]
	films	metronidazole [181]
	implant	secnidazole/doxycycline [161]
	microspheres	metro, mino, ciprofloxacin [162] chlorhexidine digluconate [163]
	nanoparticles	methylene blue [164] minocycline/arestin [165]
PCL	microfibers	metronidazole [173]
	gels membrane	metronidazole [182] ZnO [170] tetracycline [183]
Poloxamer	nanoemulsion hydrogel	quercetin [175] mebeverini hydrochloridum [177]
Ethylene-vinyl acetate (EVA)	fibers	tetracycline [184]

## 4. Directions of Development of Polymer-Based Carriers and Outlook in Dental Administration

The search for new drug delivery methods and outlook in dental administration are research areas that often require a multidisciplinary scientific approach involving collaboration between polymer chemists, drug formulation technologists, and dentists. Despite the ongoing development of dental carriers for topical drug delivery, unmet needs remain in this area to develop drug dosage forms that release the active substance in a modified manner under dynamic oral conditions to improve therapeutic strategies and patient convenience. Due to the use of polymers, it has become possible to deliver drugs in a prolonged or controlled manner, and the designed new carriers of active substances, as compared to conventional dosage forms, guarantee additional therapeutic benefits, i.e., localization of the substance’s action in the oral cavity to the inflammation-changed site, reduction or absence of systemic adverse effects of the substance, the aforementioned modification of the drug’s duration of action, adhesion to the site of application, additionally the possibility of reducing the frequency of drug dosing, ease of administration of the drug dose and, if necessary, discontinuation of therapy. However, there are some aspects related to the use of polymers as carriers of the active substance in the treatment of oral diseases that should be considered in the future in determining the direction of development of drug dosage forms and materials for dental applications, i.e.,

- The search for new biodegradable polymers for the development of carriers that release the substance in the oral cavity,
- The inclusion of mucoadhesive polymers that provide additional lubrication and physical protection of the ulcerated oral mucosa and alleviate the symptoms of ongoing inflammation,
- The use of multifunctional polymers, which, in addition to the basic function of an pharmaceutical excipient, may have additional properties such as pharmacological activity,
- The use of in situ sensitive polymers, i.e., temperature changes (poloxamers, PVA, cellulose derivatives, alginate, gellan), pH changes (Carbopol^®^, chitosan), resulting in stimuli-responsive drug carriers,
- Design of carriers tailored to the place of application in an individual dose obtained, for example, by means of 3D technology, electrospinning, realizing the idea of personalized therapy, and making it possible to obtain an effective therapeutic concentration even with a small dose of the drug,
- The administration of biologic drugs, which are difficult to deliver using conventional dosage forms and are currently almost exclusively limited to systemic administration.

Newly designed and synthesized macromolecular compounds for dental applications are expected to have properties beneficial to oral health [185]. This is why we are looking for the so-called tailor-made polymers, compounds with relatively complex structures, which is often associated with the complexity of their synthesis, low homogeneity of properties in individual batches (high dispersion), and the need to develop a course of the synthesis process allowing obtaining them in a reproducible manner. It is now emerging as possible to control certain aspects of a polymer’s structure, as well as its chemical composition, including the orientation of specific functional groups interacting with the drug, during the synthesis process to tailor its physicochemical, mechanical, and biological properties, thus producing materials suitable for the desired biological application, but for synthetic polymers, often without biodegradability characteristics [186,187]. Increasing the biodegradability of synthetic polymers is possible by introducing grouping into their backbone, i.e., ester, orthoester, amide, urea, or urethane [160]. In addition, when designing a drug dosage form based on synthetic macromolecular compounds, further processing of the polymer in the technological process must also be taken into account. This requires assessing the impact of all excipients and APIs used in the properties of the obtained carrier, particularly its biocompatibility. Therefore, the trend of using natural polymers or their modifications to design drug formulations is gaining importance. Another possible strategy is to develop materials consisting of natural and synthetic blocks, ultimately combining the beneficial properties of the natural polymer, i.e., biodegradability and biocompatibility, and the mechanical properties of a synthetic polymer [186,188].

The prolonged contact time of the drug with the body tissue can significantly improve the clinical effect of many substances used in the treatment of oral lesions. The use of mucoadhesive polymers in dental dosage forms allows the residence time of the drug formulation to be extended at the action site, thus ensuring a high concentration gradient of the released substance, often protecting it from enzymatic degradation, improving its absorption, while reducing the frequency and ease of carrier application. Therefore, it is advisable to use anionic polymers as components of mucoadhesive formulations, i.e., carboxymethyl cellulose sodium salt and alginates, pectin, xanthan gum, and hyaluronic acid interacting with mucus by forming hydrogen bonds between the carboxyl groups of the polymer and the hydroxyl groups of the mucus glycoproteins as well as the mucoadhesive cationic polymers, i.e., chitosan, having amino groups that interact with anionic substructures of sialic acid residues in the mucus layer [11,13]. The trend of designing mucosal materials and carriers for application to oral mucosa obtained by the electrospinning method is favorably evaluated. After application, these materials and carriers show a significant reduction of the ulceration area in the treatment of oral lichen planus (OLP). In effect, they are a clinically positively evaluated new dosage form of active substance application [28,189]. Oral ulcers and lesions cause pain, which can be reduced by the use of polymer-based mucosal carriers because they provide an additional protective barrier against mechanical stimulation.

The use of multifunctional polymers in the design of materials and carriers for application in dentistry, i.e., hyaluronic acid, chitosan, collagen, and gelatin, or in situ sensitive polymers, i.e., poloxamer and gums. In addition to their primary function as excipients, they allow for the exploitation of additional properties of polymers, providing a wide range of diverse functions in carriers, from the synergism of action with the active substance, promotion of osteogenesis, inhibition of bacterial biofilm formation, hemostatic action, acceleration of periodontal tissue regeneration, to sensitivity to the conditions of the application site [185]. The treatment of periodontitis often requires drug delivery inside of a pocket. In this case, the ideal solution, in addition to the use of multifunctional or stimuli-responsive polymers, is the ability to produce a carrier in any form, size, or shape tailored to the area of the site of administration, realizing the idea of personalized therapy. Three-dimensional (3D) printing as a technology for the preparation of carriers with an incorporated active substance in the desired concentration under office conditions seems to be an achievable novelty in the near future, with an individually adjusted API dose as a form of therapy, in particular in pocket applications. Flexibility in application and dosage is also provided by the use of nanofibers derived from multifunctional polymers in the prevention and treatment of alveolar bone inflammation, which is known as alveoalgia, a painful condition caused by a lack of blood clot at the site of tooth extraction [28,30]. Electrospun nanofibers carriers can act as protective shields, preventing blood clot loss or protecting the underlying bone and nerves and ultimately providing pain relief. Nanofiber engineered patches may also provide a topical anticancer therapy for oral squamous cell carcinoma or precancerous lesions when used with potential drugs including histone deacetylase (HDAC) inhibitors, i.e., hydroxamic acid suberoylanilide [28].

The administration of biologic drugs in traditional dosage forms serves as a challenge in designing active substance carriers in dentistry. Some biologic drugs, e.g., keratinocyte growth factor, monoclonal antibodies directed against pro-inflammatory cytokines, anti-TNF-α antibodies, and gene therapy, show potential in the treatment of oral disorders [190] and can be effective if delivered directly to the target site. It is indicated that the delivery of monoclonal antibodies in topical carriers would improve the treatment options for inflammatory disorders, i.e., OLP (oral lichen planus) or RAS (recurrent aphthous stomatitis). However, in order to effectively deliver the drug, the developed carrier should protect the biological substances from the enzymatic environment, which allows the use of nanocarriers, multifunctional polymers, i.e., chitosan, sodium alginate, carboxymethyl cellulose, and thiomers, which themselves exhibit poor enzyme inhibitory properties, or the use of polymer-inhibitor conjugates, i.e., chitosan–EDTA [1].

The discussed aspects of developing polymeric materials and active substance carriers may increase the number of preparations available to patients, which is in line with the expected growing demand for innovative technologies in dentistry.

## Figures and Tables

**Table 1:** An overview and characteristic of topical dental administration.

Characteristics	Oral Cavity	Periodontal Pocket Depth
Administration area/size of the administration area	surface area of periodontal pockets 50–200 cm^2^ [14], 100 cm^2^ [13]	>4 mm during an inflammatory state [17]
Type of covering epithelium	keratinized [7,13]	non-keratinized [13]
Thickness of epithelium	500–800 µm [11]	100–600 µm [13]
Exchange/flow of saliva/fluid in the gingival groove	0.5–2 L/day [8]	3–137 µL/h [13,18]
Desired characteristics of the drug carrier	adhesion, moldability, and adaptability to the application site (plasticity), resistance to smearing, patient self-application, biodegradability, modified release [7]	fitting into the application site, penetration into tissue, formation of physical bonds with tissue, resistance to smearing, adequate retention time, ease of application, biodegradability, modified release [19]
Polymeric carriers of active substance applied	rinses, films [20], gels [21], bioadhesive tablets, matrices, scaffolds	fibers, microspheres [22], implants, gels, in situ gels, nanocarriers (nanoparticles, nanospheres)

**Table 2:** Natural polysaccharides used in carriers dedicated to topical application in dentistry.

Polymer	Carrier	Drug
Chitosan	gels	VEGF [32] atorvastatin [33] thymol [34] metronidazole/vancomycin [35] lidocaine [36]
	films	*Acmella oreacea* [20] metformini hydrochloridum [37] pentoxifylline [38]
	membranes	extract from *Garcinia mangostana* [39] without substance [40] without substance [41]
	microspheres microparticles	ornidazole/doxycycline [22] tetracycline hydrochloride [42] metronidazole [43]
Hyaluronic acid Hyaluronate	membranes matrices	without substance [44] L-PRF [45] rhBMP9 [46]
	gels	0.2% [4]
Gums Pectins	film sponge matrix	triamcinolone acetonide [47] without substance [48,49] lidocaine [10]

**Table 3:** Peptides used in carriers dedicated to topical application in dentistry.

Polymer	Carrier	Drug
Gelatin	implant (Periochip^®^)	chlorhexidine digluconate [110]
	hemostatic sponge	Gelfoam^®^ without substance [111] Gelatamp Ag ions [112]
Collagen	membranes implant (PerioCol™-CG)	without substance [113] chlorhexidine digluconate [114]

**Table 4:** Semi-synthetic cellulose derivatives used in topically administered carriers in dentistry.

Cellulose Ethers	R Groups
Methylcellulose	H, CH_3_
Ethylcellulose	H, CH_2_CH_3_
Hydroxyethyl methylcellulose	H, CH_3_, [CH_2_CH_2_O]_n_H
Hydroxypropyl cellulose	H, [CH_2_CH(CH_3_)O]H
Carboxymethyl cellulose	H, CH_2_COONa

**Table 5:** Semi-synthetic cellulose derivatives used in topically administered carriers in dentistry.

Polymer	Carrier	Drug
HPMC	sponge	curcumin [138]
	film	fluticasone propionate [139] satranidazole [140] ornidazole/dexamethasone [141] metronidazole [38]
	mucoadhesive tablet	chlorhexidine digluconate [142] miconazole [143]
CMC	film	imiquimod [144] allantoin [136]
CMCNa	gel matrix	satranidazole [145] metronidazole [137]
HPC	film	dibucaine [146] tetracaine/ofloxacin [147]
EC	matrix	lidocaine/triamcinolone [148]

## Data Availability

All the data is available within the manuscript.
